# Isoliquiritigenin as a Neuronal Radiation Mitigant: Mitigating Radiation-Induced Anhedonia Tendency Targeting Grik3/Grm8/Grin3a via Integrated Proteomics and AI-Driven Discovery

**DOI:** 10.3390/ph18091307

**Published:** 2025-08-30

**Authors:** Boyang Li, Suqian Cheng, Han Zhang, Bo Li

**Affiliations:** 1 School of Interdisciplinary Science, Beijing Institute of Technology, Beijing 100081, China; boyangli@bit.edu.cn (B.L.); cheng_su_qian@163.com (S.C.); 3120232057@bit.edu.cn (H.Z.); 2 Beijing Key Laboratory for Separation and Analysis in Biomedicine and Pharmaceuticals, Beijing Institute of Technology, Beijing 100081, China; 3 School of Medical Technology, Beijing Institute of Technology, Beijing 100081, China

**Keywords:** isoliquiritigenin, radiation mitigation, glutamate homeostasis, synaptic plasticity

## Abstract

**Background/Objectives**: Radiotherapy can cause severe and irreversible brain damage, including cognitive impairment, increased dementia risk, debilitating depression, and other neuropsychiatric disorders. Current radioprotective drugs face limitations, such as single-target inefficacy or manufacturing hurdles. Isoliquiritigenin (ISL), a natural flavonoid derived from licorice root, exhibits broad bioactivities. It exhibits anti-inflammatory, anti-cancer, immunoregulatory, hepatoprotective, and cardioprotective activities. This study aimed to elucidate ISL’s neuronal radiation mitigation effects and key targets. **Methods**: In vitro and in vivo models of radiation-induced neuronal injury were established. ISL’s bioactivities were evaluated through cellular cytotoxicity assays, LDH release, ROS, ATP, glutamate, and GSH levels. In vivo, ISL’s radiation mitigation effect was evaluated with sucrose preference test, IL-β level, histopathological analysis, and Golgi-Cox staining analysis. Proteomics, pathway enrichment, and ensemble models (four machine learning models, weighted gene co-expression network, protein–protein interaction) identified core targets. Molecular docking and dynamic simulations validated ISL’s binding stability with key targets. **Results**: ISL attenuated radiation-induced cellular cytotoxicity, reduced LDH/ROS, restored ATP, elevated GSH, and mitigated glutamate accumulation. In rats, ISL alleviated anhedonia-like phenotypes and hippocampal synaptic loss. ISL also significantly suppressed radiation-induced neuroinflammation, as evidenced by reduced levels of the pro-inflammatory cytokine IL-1β. Proteomic analysis revealed that ISL’s main protective pathways included the synaptic vesicle cycle, glutamatergic synapse, MAPK signaling pathway, SNARE interactions in vesicular transport, insulin signaling pathway, and insulin secretion. Grm8, Grik3, and Grin3a were identified as key targets using the integrated models. The expression of these targets was upregulated post-radiation and restored by ISL. Molecular docking and dynamic simulations indicated that ISL showed stable binding to these receptors compared to native ligands. **Conclusions**: ISL demonstrates multi-scale radiation mitigation activities in vitro and in vivo by modulating synaptic and inflammatory pathways, with glutamate receptors as core targets. This work nominates ISL as an important natural product for mitigating radiotherapy-induced neural damage.

## 1. Introduction

Radiotherapy (RT) is essential for treating brain and neck tumors but damages healthy brain tissue [1]. Around 5–43% of meningioma patients experience symptomatic edema post-SRS, of which 56% require hospitalization and 12.5% prove fatal [2]. RT also frequently induces cognitive decline, with deficits particularly evident in memory, attention, and executive function [3]. Furthermore, RT also cause life-threatening complications such as cerebral radiation necrosis [4]. Although advanced techniques such as proton therapy reduce low-dose exposure per fraction, their technical limitations often necessitate multiple treatment sessions [5]. The multiple irradiations can lead to accumulation of radiation damage, resulting in radiation-induced cognitive dysfunction (RICD), characterized by mild deficit or severe dementia, including progressive deficit in executive function, episodic memory, information processing speed, and spatial orientation, all of which substantially impact patients’ quality of life [6].

Mechanistically, brain radiation therapy (RT) damages multiple central nervous system (CNS) cell types (e.g., astrocytes, microglia, oligodendrocytes, endothelial cells, neural stem cells), triggering early neuroinflammatory responses [7]. This inflammatory response promotes inflammatory factor release and cellular infiltration, disrupting the neural microenvironment and inducing cellular senescence, thus ultimately driving radiation-induced cognitive decline [8].

Current pharmacological interventions for radiation-induced neural damage remain limited [9]. Conventional agents, including corticosteroids (e.g., dexamethasone for acute inflammation [10]), antioxidants (e.g., amifostine [11]), and growth factors (e.g., EGF [12]), primarily address single pathways such as oxidative stress or inflammation. Although nanomedicines offer potential for multi-target action and improved biodistribution, they face challenges in scalable manufacturing and unresolved long-term toxicity concerns [13]. Consequently, most clinical options lack effective strategies against the interconnected cascade of low toxicity, minimal side effects, and multiple targets underlying RICD [14].

Isoliquiritigenin (ISL) is a natural bioactive flavonoid component isolated from the roots of licorice, including Glycyrrhiza uralensis, Mongolian glycyrrhiza, Glycyrrhiza glabra, and so on [15]. Licorice is not only a common food additive but also a fundamental herb in Traditional Chinese Medicine (TCM). Licorice has been reported to exhibit diverse pharmacological effects and may be used as an adjunctive therapy for peptic ulcers, alleviating respiratory tract inflammation, and demonstrates anti-inflammatory, antioxidant, and immunomodulatory properties [16]. In addition, ISL is found in various agricultural products and foods, such as soybeans and onions [17]. Extensive research has demonstrated that ISL possesses a broad spectrum of pharmacological properties [18]. It is reported to exhibit notable anti-inflammatory effects [19] and demonstrate antimicrobial and potent antioxidant activities [20,21]. Studies have also highlighted its hepatoprotective and cardioprotective effects [22]. ISL has been shown to modulate various signaling pathways to induce apoptosis and inhibit tumor proliferation [23]. Finally, it has also been found to alleviate postprandial hyperglycemia effects by inhibiting α-amylase [24].

Previously, we have demonstrated that Dragon’s Blood (DB), a non-toxic, brown-red resin extracted from *Dracaena cochinchinensis* (Lour.) (S. C. Chen, China), exhibits radiation mitigation effects in liver [25]. Extensive studies have also demonstrated its therapeutic potential, including cardiovascular protection, promotion of blood circulation, and resolution of blood stasis, as well as anti-inflammatory and antioxidant effects [26,27]. Employing network pharmacology, we identified ISL as a primary natural compound responsible for the radiation mitigation activity of DB. Herein, in order to investigate ISL’s neuronal radiation mitigation effect and identify its molecular targets, an integrated experimental–computational ensemble framework was employed. In vitro studies demonstrated that ISL effectively mitigated radiation-induced damage, significantly restoring cell viability, reducing membrane damage, and restoring cellular ATP level. Furthermore, ISL also counteracted the radiation-induced elevation of glutamate and maintained antioxidant capacity (GSH levels). In vivo, ISL administration alleviated radiation-triggered anhedonia-like behaviors in rats. This protective effect was associated with preserved hippocampal structural integrity, including the maintenance of critical dendritic spine morphologies, and reduced histopathological neuronal damage. ISL treatment also suppressed radiation-induced neuroinflammation (IL-1β elevation) and alleviated the radiation-induced decrease in PSD-95. These findings demonstrate ISL’s potent radiation mitigation efficacy across cellular, histological, and behavioral levels.

Given that proteomics can comprehensively reflect the regulation of ISL’s radiation mitigation, 4D-DIA proteomic profiling of rat brain tissues was conducted. Pathway enrichment revealed that ISL’s radiation mitigation is mainly related to synaptic vesicle cycle, the glutamatergic synapse, the MAPK signaling pathway, SNARE interactions in vesicular transport, the insulin signaling pathway, and insulin secretion. Weighted gene co-expression network analysis (WGCNA), four ensembled machine learning models, and the protein–protein interaction (PPI) network further prioritized 12 key proteins. Among these, glutamate receptors Grik3, Grm8, and Grin3a were identified as ISL’s key radiation mitigation targets based on functional annotation. Molecular docking predicted strong binding affinity between ISL and these receptors, and molecular dynamics simulations further supported the stability of these ISL–receptor complexes. In summary, this work establishes ISL as a potent natural radiation protectant that modulates glutamate homeostasis and synaptic plasticity primarily through targeting glutamate receptors Grik3, Grm8, and Grin3a.

## 2. Results

### 2.1. Isoliquiritigenin’s Protection Effect Against Radiation-Induced Damage at the Cellular Level

**Figure 1:**
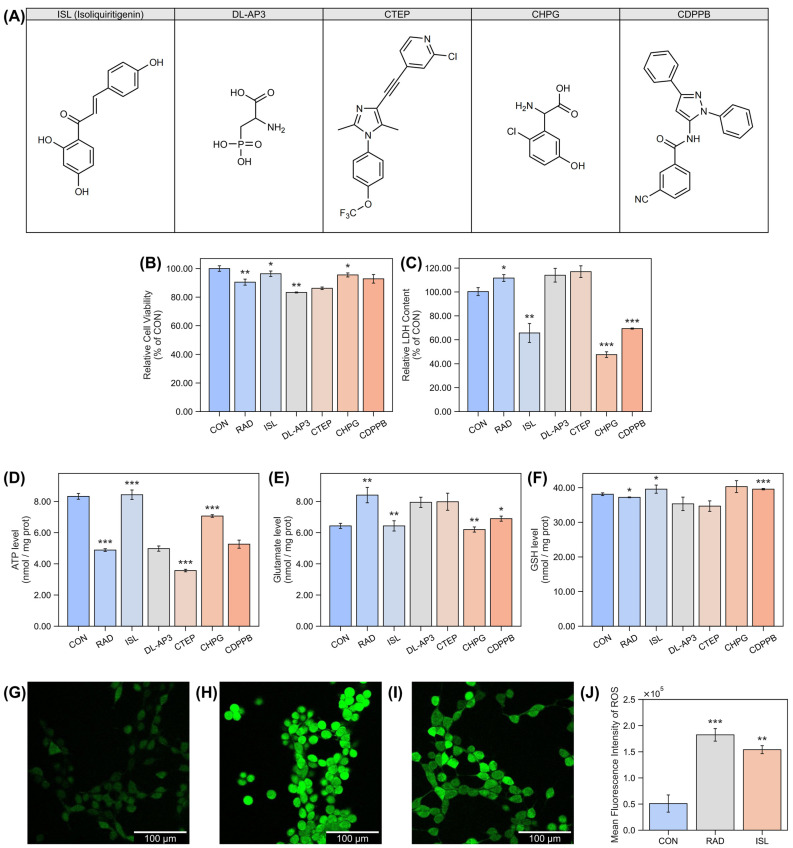
Isoliquiritigenin’s protection effect against radiation-induced damage at the cellular level. (**A**) Chemical structure of ISL, DL-AP3, CTEP, CHPG, and CDPPB. (**B**) Effect of different compounds on cell viability. (**C**) Effect of different compounds on LDH secretion. (**D**) Effect of different compounds on ATP level. (**E**) Effect of different compounds on glutamate level. (**F**) Effect of different compounds on reduced glutathione (GSH) level. Boxplot indicates mean ± standard deviation. * *p*-value < 0.05; ** *p*-value < 0.01; *** *p*-value < 0.001. RAD was compared to CON group. ISL, DL-AP3, CTEP, CHPG, and CDPPB were compared to RAD group. (**G**–**I**) Fluorescence microscopy images visualizing intracellular reactive oxygen species (ROS) levels in CON (**G**), RAD (**H**), and ISL (**I**). (**J**) Mean fluorescence intensity of ROS.

To evaluate the cytoprotective potential of ISL against radiation-induced cellular damage, HT22 mouse hippocampal neuronal cells were employed. For comparative analysis, four known metabotropic glutamate receptor modulators were selected (Figure 1A): DL-AP3, a glutamate receptor antagonist [28]; CTEP, a mGluR5 negative allosteric modulator (NAM) [29]; CHPG, a mGluR5 agonist [30]; and CDPPB, a mGluR5 positive allosteric modulator (PAM) [31]. Cellular responses were assessed through viability, membrane integrity, bioenergetic status, excitotoxicity, and antioxidant capacity assays.

To determine radioprotective effects at the cellular level, cell viability was quantified using CCK-8 assay, which measures metabolic activity as an indicator of live cells (Figure 1B). To ensure each compound’s protective effect without inducing any cellular toxicity, CCK-8 assay was employed to determine each compound’s selected concentration (Figure S1). Radiation exposure significantly reduced cell viability to 90.5 ± 2.1% of control levels. Among mGluR modulators, the agonist CHPG increased viability to 95.5 ± 1.4%, while the PAM CDPPB showed no significant effect (92.8 ± 3.0%). Conversely, the NAM CTEP exhibited no protection (86.2 ± 0.9%), and the antagonist DL-AP3 exacerbated damage (83.3 ± 0.3%). ISL demonstrated significant protection, restoring viability to 96.4 ± 1.9%.

Membrane integrity was evaluated via an LDH release measurement, where extracellular LDH indicates plasma membrane damage (Figure 1C). Radiation significantly increased LDH release to 111.7 ± 2.9% of control levels. The agonist CHPG and positive allosteric modulator CDPPB showed robust protection, reducing release to 47.7 ± 2.4% and 69.3 ± 0.5%, respectively. No significant attenuation occurred with DL-AP3 (114.0 ± 5.7%) or CTEP (117.0 ± 4.9%). ISL markedly attenuated LDH release to 66.0 ± 8.0%.

Bioenergetic status was assessed through ATP quantification, reflecting mitochondrial function and cellular energy reserves, as shown in Figure 1D. Radiation severely depleted ATP to 58.6 ± 1.0% of CON (RAD: 4.8 nmol/mg prot, CON: 8.3 nmol/mg prot). The agonist CHPG partially restored ATP to 84.8 ± 1.0% of CON (CHPG: 7.1 nmol/mg prot), while CDPPB (5.3 nmol/mg prot) and DL-AP3 (5.0 nmol/mg prot) showed no significant effects (63.2 ± 3.1% of CON and 59.8 ± 2.0% of CON). The NAM CTEP (3.6 nmol/mg prot) exacerbated depletion (42.8 ± 1.0%). In contrast, ISL completely restored ATP to 101.2 ± 3.6% (8.4 nmol/mg prot).

Excitotoxic stress was evaluated by measuring intracellular glutamate, where elevated levels indicate neuronal overexcitation, as shown in Figure 1E. Radiation significantly increased glutamate to 130.7 ± 7.7% of CON (RAD: 8.4 nmol/mg prot; CON: 6.4 nmol/mg prot). Significant reductions were achieved by the agonist CHPG (96.4 ± 2.6%, 6.2 ± 0.2 nmol/mg prot) and positive allosteric modulator CDPPB (107.2 ± 2.6%, 6.9 ± 0.2 nmol/mg prot). DL-AP3 (123.5 ± 5.1%, 7.9 ± 0.3 nmol/mg prot) and CTEP (124.1 ± 8.5%, 8.0 ± 0.5 nmol/mg prot) showed no significant modulation. ISL normalized glutamate to baseline levels (100.0 ± 3.3%, 6.4 ± 0.3 nmol/mg prot).

Antioxidant capacity was determined via reduced glutathione (GSH) quantification, a critical endogenous free radical scavenger. As shown in Figure 1F, significant radiation-induced alteration occurred (37.2 ± 0.1 nmol/mg prot vs. CON: 38.1 ± 0.4 nmol/mg prot). The positive allosteric modulator CDPPB significantly elevated GSH to 103.9 ± 0.5% (39.6 ± 0.2 nmol/mg prot). ISL also significantly elevated GSH to 103.9 ± 3.1% (39.6 ± 1.2 nmol/mg prot). DL-AP3 (35.3 ± 1.9 nmol/mg prot), CTEP (34.7 ± 1.5 nmol/mg prot), and CHPG (40.3 ± 1.7 nmol/mg prot) showed no significant modulation.

To further study the antioxidant ability of ISL, the DCFH-DA probe was used for ROS fluorescence imaging. The fluorescence intensity is proportional to the level of intracellular ROS (Figure 1G–J). After radiation, the fluorescence intensity of the cells increased significantly by 257.9%. In comparison, cellular fluorescence intensity treated with ISL was decreased by 15.6% compared to cells without ISL. The effect sizes and confidence intervals of the significance test were shown in Table S2. The above-mentioned results indicated that ISL could effectively reduce the radiation-induced oxidative stress.

### 2.2. Isoliquiritigenin’s Radiation Mitigation Effect Against Radiation Damage at the Animal Level

To investigate the effect of ISL against radiation-induced damage at the animal level, a Wistar rat model of whole-brain irradiation injury (30 Gy [32,33,34]) was established. The ISL group received a daily intragastric administration of 20 mg/kg fresh ISL aqueous suspension. A comprehensive analysis encompassing histological examination, quantification of key molecular markers, and detailed assessment of neuronal morphology was conducted.

Specifically, hematoxylin and eosin (H&E) staining was performed to evaluate gross structural integrity in the hippocampus, while molecular analyses quantified synaptic integrity via PSD-95 expression and neuroinflammation via IL-1β levels. Furthermore, dendritic spine density and morphology were analyzed to assess synaptic plasticity and neuronal connectivity changes.

**Figure 2:**
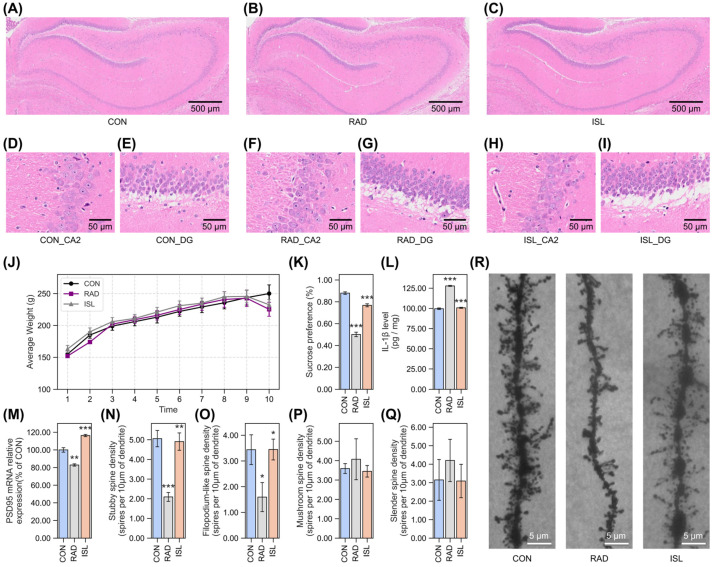
ISL’s radiation mitigation effect against radiation-induced damage at the animal level. (**A**,**D**,**E**) H&E staining results of the rat hippocampi in the CON group (**A**), CA2 region (**D**), and gyrus region (**E**). (**B**,**F**,**G**) H&E staining results of the rat hippocampi three days post-radiation (**B**), CA2 region (**F**), and gyrus region (**G**). (**C**,**H**,**I**) H&E staining results of the rat hippocampi three days post-radiation with administration of ISL (**C**), CA2 region (**H**), and gyrus region (**I**). (**J**) Average weight of rats in different groups. (**K**) Rat sucrose preference behavior results. (**L**) The relative level of IL-1β in rat brains. (**M**) The relative mRNA levels of PSD-95 in rat brains. (**N**) Quantitative analysis of the density of stubby spine in rat hippocampi. (**O**) Quantitative analysis of the density of filopodium-like spine in rat hippocampi. (**P**) Quantitative analysis of the density of mushroom spine in rat hippocampi. (**Q**) Quantitative analysis of the density of slender spine in rat hippocampi. (**R**) Morphology of the dendrites 3 days post-radiation (1000×). For (**K**–**Q**), the boxplot indicates mean ± standard deviation (*n* = 3). * *p*-value < 0.05; ** *p*-value < 0.01; *** *p*-value < 0.001. RAD was compared to the CON group. ISL was compared to the RAD group.

To visualize radiation-induced structural alterations and ISL’s radiation mitigation effects in the hippocampal formation, H&E staining was performed on rat brain sections. Significant histological alterations were observed in the hippocampus three days after irradiation. In the CON group (Figure 2A), the hippocampus exhibited a normal histological architecture characterized by distinct cellular layers and intact neuronal morphology, which was corroborated by a low histopathological score averaging 0.4. In contrast, the radiation-exposed group (Figure 2B) displayed substantial damage, manifesting as nuclear pyknosis, cytoplasmic eosinophilia, and a general disruption of tissue organization, resulting in a significantly elevated average score of 2.8. However, treatment with ISL (Figure 2C) markedly attenuated these radiation-induced pathological changes, effectively preserving the overall integrity of the hippocampal structure compared to the RAD group, as reflected in a notably reduced average score of 1.2. Detailed examination of specific hippocampal subregions further elucidated this protection. Within the CA2 region, radiation exposure (Figure 2F) resulted in a marked reduction in neuronal cell density and increased cellular debris when compared to the control (Figure 2D), with average scores rising from 0.3 in CON to 2.7 in RAD. In contrast, the ISL-treated group (Figure 2H) showed significantly less pronounced damage, maintaining a cellular density and organization much closer to control levels and achieving an average score of 1.1. Similarly, in the dentate gyrus (DG), the RAD group (Figure 2G) exhibited significant neuronal loss and increased vacuolation relative to the CON group (Figure 2E), reflected in an increase in average scores from 0.5 to 2.9. This radiation-induced damage was notably mitigated by ISL (Figure 2I), which demonstrated better preservation of neuronal morphology and reduced vacuolation, with a significantly decreased average score of 1.3.

To further evaluate the radiation-induced effect and the radiation mitigation effect of ISL across the entire hippocampal formation, quantitative neuronal counts on hemibrain sections were conducted. The average number of neurons in the CON group was 2414 ± 92, while the RAD group showed a significant reduction, with an average count of 1970 ± 21. In contrast, ISL treatment significantly restored neuronal numbers, yielding an average count of 2674 ± 154, indicating a notable mitigation of radiation-induced neuronal loss.

There was not a significant difference between groups in the body weights before radiation. On the third day after irradiation, the average body weights of the CON group, RAD group, and ISL group were 250.1 ± 13.6 g, 225.3 ± 11.0 g, and 232.5 ± 8.0 g. The average body weight of the RAD and ISL groups slightly decreased compared to that of the CON group (Figure 2J). A sucrose preference test was conducted to assess rats’ anhedonia [35]. The sucrose preference rate of the RAD group was significantly decreased compared to the CON group (Figure 2K). In contrast, the sucrose preference rate in the ISL groups increased significantly by 53.3%, suggesting that ISL prevents radiation-induced anhedonia-like behavior.

To evaluate the impact of radiation and ISL on neuroinflammation, the relative level of the pro-inflammatory cytokine IL-1β was measured (Figure 2L). A significant elevation in IL-1β levels was observed in the RAD group (128.3% ± 0.6%, 127.9 ± 0.6 pg/mg) compared to the CON group (100.0% ± 0.9%, 99.63 ± 0.9 pg/mg), indicating a 28.3% increase. Treatment with ISL significantly suppressed this radiation-induced inflammatory response, reducing IL-1β levels to 101.1% ± 0.6% (100.7 ± 0.6 pg/mg). This represents a 27.2% decrease compared to the RAD group (% of the CON).

To assess changes in brain synaptic integrity associated with radiation damage and ISL, the relative mRNA expression levels of the synaptic marker PSD-95 were quantified, (Figure 2M). Radiation induced a significant decrease in PSD-95 expression (82.9% ± 1.3%) relative to the CON group (100.0% ± 2.5%), representing a 17.1% reduction. Administration of ISL partially restored PSD-95 expression levels (116.3% ± 1.3%), which were significantly increased (by 33.4%) compared to the RAD group (% of the CON).

To investigate the effects on synaptic plasticity, which is closely reflected by dendritic spine density and morphology, quantitative analysis of different spine types was performed on dendrites. Spine density is correlated with synaptic strength and connectivity. Four distinct morphological types were assessed: stubby spines, filopodium-like spines, mushroom spines, and slender spines. Stubby spine density (Figure 2N) was significantly decreased by radiation exposure (2.094 ± 0.224 spines/10 μm of dendrite) compared to CON (5.056 ± 0.414 spines/10 μm), reflecting a substantial 58.6% reduction. ISL significantly ameliorated this loss, resulting in a density of 4.905 ± 0.441 spines/10 μm, which was 134.2% higher than the RAD group and approaching control levels (97.0% of CON). Similarly, radiation caused a significant decline in filopodium-like spine density (1.596 ± 0.564 spines/10 μm) relative to controls (3.440 ± 0.579 spines/10 μm), equating to a 53.6% decrease (Figure 2O). ISL significantly reversed this, increasing density to 3.446 ± 0.404 spines/10 μm. This level was 115.9% higher than the RAD group and corresponded to 100.2% of the control value. In contrast, the density of mushroom spines (Figure 2P) showed no significant difference among the groups (CON: 3.593 ± 0.255; RAD: 4.072 ± 1.056; ISL: 3.452 ± 0.298 spines/10 μm). Slender spine density (Figure 2Q) exhibited a trend towards a decrease in the RAD group (4.203 ± 1.143 spines/10 μm) compared to CON (3.151 ± 1.105 spines/10 μm), and a partial numerical recovery with ISL (3.091 ± 0.904 spines/10 μm).

To further characterize the impact of radiation and ISL on neuronal structure, the morphology of dendrites was examined under high magnification (1000×) on the third day post-irradiation (Figure 2R). Consistent with the quantitative spine data, the RAD group displayed a reduction in dendritic complexity, characterized by fewer and shorter branches compared to the CON group. The ISL group exhibited a dendritic arborization pattern that was more similar to CO, showing better preservation of dendritic length and branching.

These findings demonstrate that ISL provides mitigation against radiation-induced hippocampal damage in rats. For the significance test, the effect sizes and confidence intervals are reported in Table S3. The radiation mitigation is evidenced by the preservation of normal histological architecture upon H&E staining, a reduction in radiation-induced neuroinflammation, maintenance of specific dendritic spine densities crucial for synaptic plasticity, and attenuation of radiation-induced dendritic atrophy.

### 2.3. Analysis of Isoliquiritigenin-Related Radiation Mitigation Mechanism Based on Proteomics

**Figure 3:**
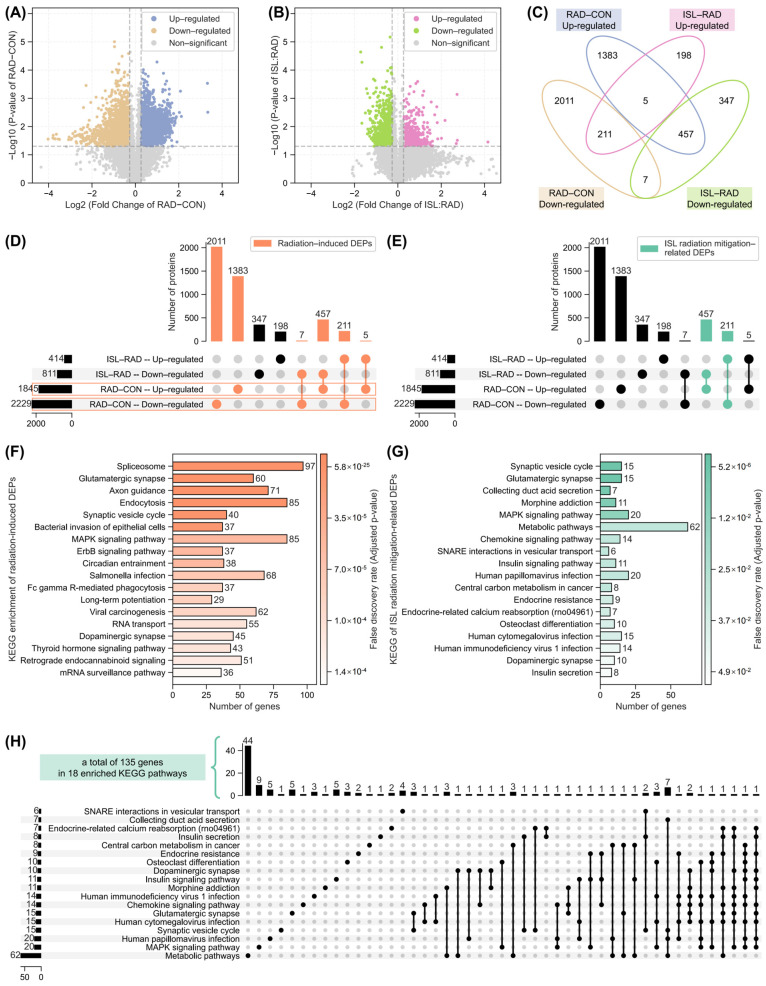
Analysis of ISL-related radiation mitigation mechanism based on proteomics. (**A**) Volcano plot depicting DEPs between the CON group and RAD group. (**B**) Volcano plot illustrating DEPs between the RAD group and ISL group. (**C**) Intersection analysis of DEPs across CON, RAD, and ISL groups. (**D**) Identification of key proteins associated with radiation-induced damage. (**E**) Identification of key proteins related to isoliquiritigenin radiation mitigation. (**F**) KEGG pathway enrichment analysis of genes corresponding to radiation-induced DEPs. (**G**) KEGG pathway enrichment analysis of genes corresponding to ISL radiation mitigation-related DEPs. (**H**) Upset plot showing the intersection of 135 genes across 18 KEGG pathways related to ISL radiation mitigation.

To elucidate the molecular mechanisms underlying the radiation mitigation effects of ISL, proteomic analysis of rat brain tissue was conducted. The principal component analysis (PCA) plot of 15 samples is shown in Figure S2. After differential expression analysis across RAD–CON and ISL–RAD (with fold change threshold 1.2 and *p*-value threshold 0.05), significant proteomic alterations following radiation exposure were revealed by volcano plot analysis (Figure 3A). A total of 1845 up-regulated and 2229 down-regulated differentially expressed proteins (DEPs) were identified in the RAD group compared to the CON group. Analysis comparing ISL–RAD identified 414 up-regulated and 811 down-regulated DEPs (Figure 3B). Intersection analysis of these DEP sets was performed using Venn and Upset plots, as shown in Figure 3C. The 1845 up-regulated and 2229 down-regulated DEPs identified in the RAD–CON comparison were designated as radiation-induced DEPs (Figure 3D). Through intersection analysis (Figure 3E), two subsets of ISL-related radiation mitigation were identified: 457 proteins that were up-regulated in RAD–CON and down-regulated in ISL–RAD and 211 proteins that were down-regulated in RAD–CON and up-regulated in ISL–RAD. These 4074 (1845 + 2229) and 668 (457 + 211) proteins were defined as radiation-induced DEPs and ISL radiation mitigation-related DEPs. A hierarchical clustering heatmap of ISL radiation mitigation-related DEPs is shown in Figure S3.

**Figure 4:**
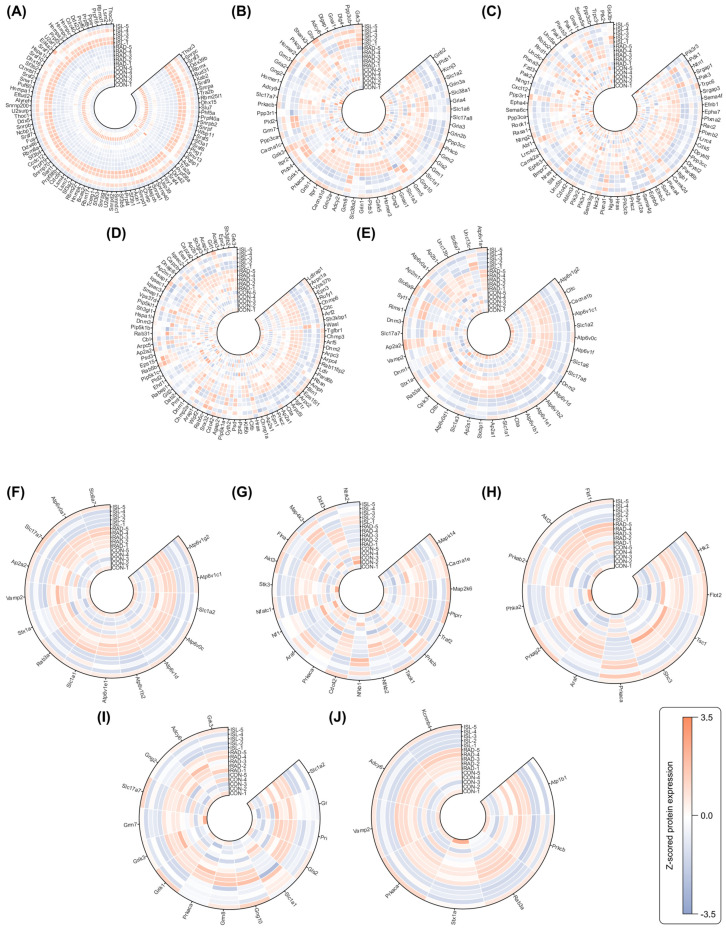
Protein expression in KEGG pathways. (**A**–**E**) proteins in KEGG pathways enriched by radiation-induced DEPs. (**F**–**J**) proteins in KEGG pathways enriched by ISL radiation mitigation-related DEPs. KEGG pathways: (**A**) spliceosome, (**B**) glutamatergic synapse, (**C**) axon guidance, (**D**) endocytosis, (**E**) synaptic vesicle cycle, (**F**) synaptic vesicle cycle, (**G**) glutamatergic synapse, (**H**) MAPK signaling pathway, (**I**) insulin signaling pathway, (**J**) insulin secretion.

KEGG pathway enrichment analysis was performed on the 4074 radiation-induced DEPs and the 668 ISL radiation mitigation-related DEPs. For radiation-induced DEPs (Figure 3F), the most significant five pathways were the spliceosome, glutamatergic synapse, axon guidance, endocytosis, and synaptic vesicle cycle. Enrichment analysis of the ISL radiation mitigation-related DEPs (Figure 3G) identified the synaptic vesicle cycle, glutamatergic synapse, MAPK signaling pathway, insulin secretion, and insulin signaling pathway. It was noted that the glutamatergic synapse, MAPK signaling pathway, and synaptic vesicle cycle pathways were significantly enriched in both the radiation-induced and ISL radiation mitigation-related sets, highlighting ISL’s radiation mitigation effect. The proteins and expression levels corresponding to these pathways are shown in Figure 4.

KEGG pathway enrichment revealed that ISL’s radiation mitigation mechanism predominantly involves the modulation of the glutamatergic synapse, MAPK signaling pathway, and synaptic vesicle cycle pathways. These findings align with the observed phenotypic effects. At the cellular level, the normalization of radiation-induced elevated intracellular glutamate and the restoration of ATP levels by ISL directly correspond to the reversal of perturbations in these key pathways. At the animal level, ISL’s significant up-regulation of PSD-95 expression (counteracting radiation-induced down-regulation), reduction in IL-1β levels (counteracting radiation-induced up-regulation, a key MAPK pathway output), and preservation of dendritic spine density and morphology provide functional validation of the proteomic-identified KEGG pathways. This multi-level concordance supports the central role of glutamatergic signaling regulation, MAPK pathway modulation, and synaptic vesicle machinery in ISL-mediated radiation mitigation.

**Figure 5:**
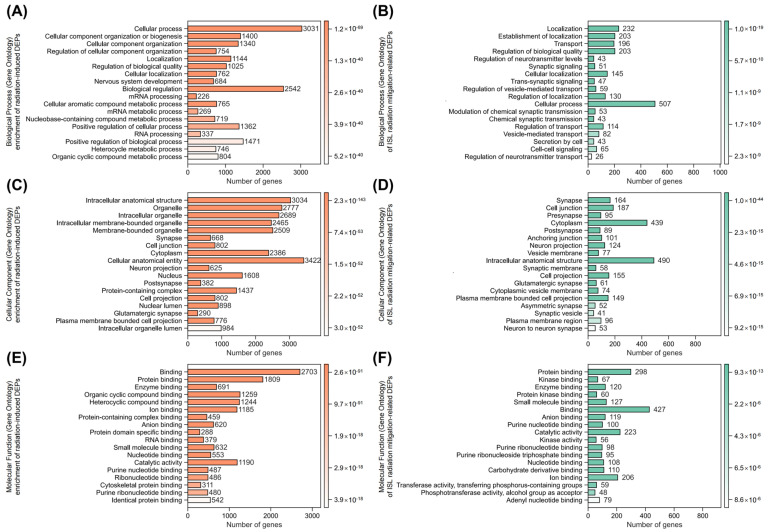
GO enrichment analysis of radiation-induced DEPs and ISL radiation mitigation-related DEPs. (**A**) Biological process (Gene Ontology) enrichment of radiation-induced DEPs. (**B**) Biological process (Gene Ontology) enrichment of ISL radiation mitigation-related DEPs. (**C**) Cellular component (Gene Ontology) enrichment of radiation-induced DEPs. (**D**) Cellular component (Gene Ontology) enrichment of ISL radiation mitigation-related DEPs. (**E**) Molecular function (Gene Ontology) enrichment of radiation-induced DEPs. (**F**) Molecular function (Gene Ontology) enrichment of ISL radiation mitigation-related DEPs.

Gene Ontology (GO) enrichment analysis was performed to characterize the functions of radiation-induced and ISL radiation mitigation-related DEPs. For radiation-induced damage (Figure 5A), key biological process included nervous system development, cellular localization, regulation of cellular component organization, and mRNA processing, reflecting disruptions in neuronal growth, intracellular trafficking, and synaptic integrity. In contrast, ISL radiation mitigation-related DEPs (Figure 5B) exhibited prominent enrichment in regulation of neurotransmitter levels, synaptic signaling, modulation of chemical synaptic transmission, and regulation of vesicle-mediated transport, implicating ISL in restoring synaptic vesicle dynamics and glutamate homeostasis.

For the cellular component in GO enrichment, radiation-induced DEPs (Figure 5C) were enriched in the synapse, neuron projection, and intracellular membrane-bounded organelle, indicating structural compromise in synaptic compartments and organelle membranes. Conversely, ISL-related radiation mitigation (Figure 5D) highlighted the pre-synapse, post-synapse, glutamatergic synapse, and synaptic membrane, pinpointing ISL’s role in preserving excitatory synapse architecture and receptor localization critical for plasticity.

For the molecular function in GO enrichment, radiation-related MFs (Figure 5E) featured protein binding, enzyme binding, and small molecule binding, suggesting the broad dysregulation of synaptic protein complexes and metabolic pathways. Under ISL’s radiation mitigation (Figure 5F), kinase binding, small molecule binding, and purine nucleotide binding were dominant, emphasizing ISL’s modulation of kinase signaling cascades (e.g., glutamate receptor phosphorylation) and nucleotide-dependent synaptic repair.

Finally, an Upset plot was generated to visualize the intersection of genes mapped to 18 KEGG pathways enriched in ISL radiation mitigation-related DEPs (Figure 3H). A core set of 135 genes that were recurrently represented across these pathways was identified, implicating them as key proteins of ISL’s radiation mitigation-related mechanism.

### 2.4. WGCNA of Co-Expression Proteins Related to Isoliquiritigenin’s Radiation Mitigation

The co-expression protein networks implicated in the radiation mitigation mechanisms of ISL were investigated through weighted gene co-expression network analysis (WGCNA). Since the radiation mitigation effect of ISL was primarily aimed at mitigating radiation-induced damage compared to the CON group, there is potential correlation between the ISL and CON groups. Therefore, a WGCNA of all three groups (*n* = 15) was considered inappropriate. Instead, separate WGCNA were conducted for the following two settings: (a) the CON and RAD group and (b) the RAD and ISL group.

**Figure 6:**
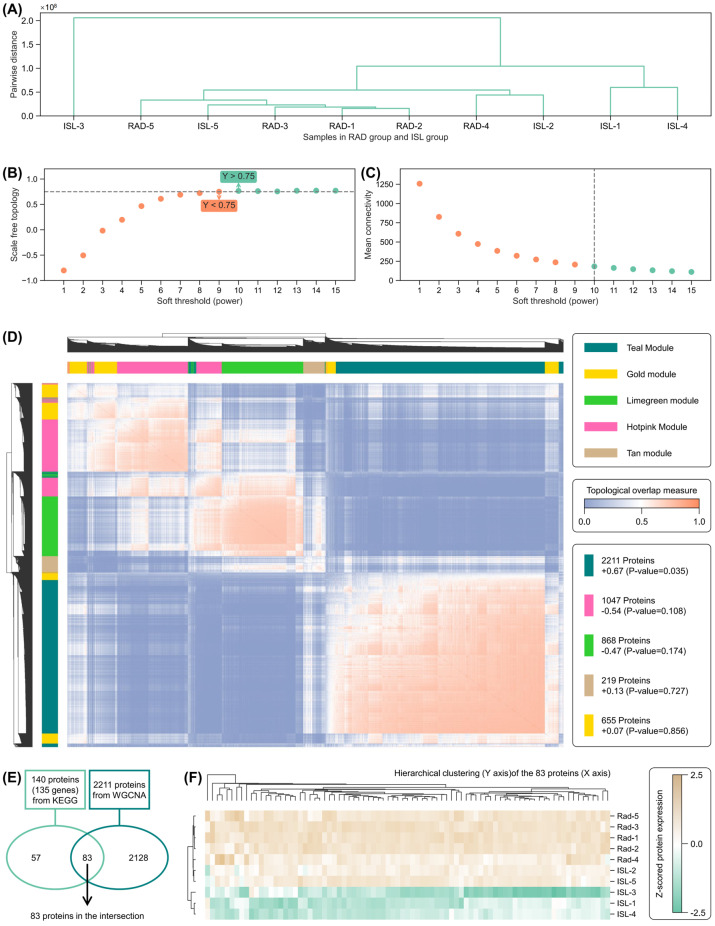
WGCNA of co-expression proteins related to ISL radiation mitigation. (**A**) Hierarchical clustering of samples in RAD and ISL group. (**B**) Scale-free topology under different soft-thresholds. (**C**) Mean connectivity under different soft-thresholds. (**D**) Topological overlap matrix dendrogram and module–trait correlations. (**E**) Intersection analysis between WGCNA-derived proteins and genes in KEGG-enriched radiation mitigation pathways. (**F**) Heatmap and hierarchical clustering for the 83 proteins across samples in RAD group and ISL group.

The initial phase of a WGCNA is hierarchical clustering and soft threshold search. For the CON and RAD groups, hierarchical clustering identified three primary subgroups: (CON-2, CON-4, CON-5), (RAD-5, RAD-3, RAD-1, RAD-2), and (CON-3, RAD-4, CON-1) (Figure S4A). Parameter search across power values from 1 to 15 failed to yield a satisfactory scale-free topology fit more than 0.75 (Figure S4B,C). In contrast, analysis of RAD and ISL groups showed ISL-3 as the most prominent outlier, followed by ISL-1 and ISL-4 (Figure 6A), with power search demonstrating that values more than 10 achieved the required scale-free topology threshold (more than 0.75) while maintaining optimal mean connectivity, leading to the selection of power of 10 as the final parameter (Figure 6B,C).

The topological overlap matrix (TOM) dendrogram and module–trait correlations are shown in Figure 6D. Five modules were identified (Teal: 2211 proteins; Gold: 1047 proteins; Lime green: 868 proteins; Hot pink: 219 proteins; Tan: 655 proteins). Module–trait correlation analysis revealed that only the Teal module exhibited a *p*-value less than 0.05.

Therefore, the 2211 proteins in Teal module were identified as key proteins related to ISL radiation mitigation by WGCNA. The intersection between 2211 proteins (in the Teal module) and 140 genes from KEGG-enriched radiation mitigation pathways are regarded as ISL radiation mitigation-related key proteins at this stage. In total, 83 proteins were found to overlap (Figure 6E). A heatmap of hierarchical clustering for these 83 proteins across 10 samples in the RAD group and ISL group is shown in Figure 6F. RAD group samples clustered together with relatively positive z-scores, while ISL group samples exhibited relatively negative expression. Overall, the clear segregation of RAD and ISL groups in the hierarchical clustering of these 83 proteins across 10 samples confirms that these proteins capture ISL’s radiation mitigation, with a distinct protein expression level.

### 2.5. Identification of Key Radiation-Induced and Isoliquiritigenin Radiation Mitigation-Related Proteins Using Four Machine Learning Models

**Figure 7:**
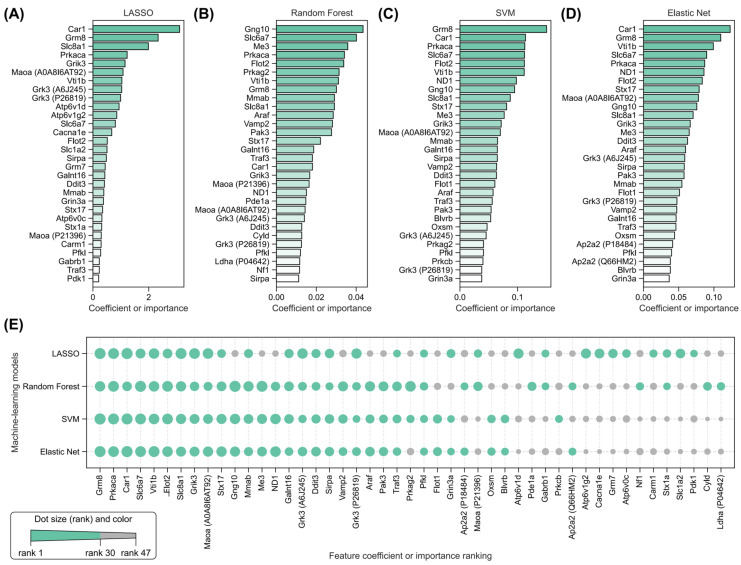
Identification of key ISL radiation mitigation-related proteins using four machine learning models. (**A**) LASSO, top 30 discriminatory proteins ranked by absolute coefficient value. (**B**) Random Forest, top 30 discriminatory proteins ranked by Gini importance (0–1 scaled). (**C**) SVM: top 30 discriminatory proteins ranked by absolute primal hyperplane coefficient value. (**D**) Elastic Net: top 30 discriminatory proteins ranked by absolute coefficient value. (**E**) Rankings across four models for the union set of 4 top 30 protein groups selected (47 proteins in total).

Four machine learning models, LASSO, Random Forest, SVM, and Elastic Net, were employed to further identify the key proteins related to ISL radiation mitigation from the above 83 proteins. For ISL radiation mitigation (ISL–RAD, Figure 7A–D), the four models independently identified top 30 discriminatory proteins (two-class classification task, to classify 5 ISL samples and 5 RAD samples). The ROC-AUC values of the four machine learning models are all 1.0 (100%). It should be noted that the objective of applying these four machine learning models is to identify the most differentially expressed proteins between the RAD and ISL groups. Given the limited sample size (*n* = 5 per group) and the variability between samples, splitting the training, validation, and testing sets is not applicable here. Under random seed 42, LASSO highlighted Car1, Grm8, Slc8a1, Prkaca, and Grik3. Random Forest prioritized Gng10, Slc6a7, Me3, Prkaca, and Flot2. SVM featured Grm8, Car1, Prkaca, Slc6a7, and Flot2. Elastic Net emphasized Grm8, Car1, Prkaca, Vtl1b, and Grk3. The top 30 proteins selected by the four models collectively involved 47 unique proteins. Their rankings of importance or coefficient values across the four models are shown in Figure 7E. The results of stability testing of the “top 30” features are included in Table S4.

### 2.6. Identification of Key Isoliquiritigenin Radiation Mitigation-Related Targets Based on Protein–Protein Interaction Networks, KEGG/GO Enrichment and Functional Annotation

**Figure 8:**
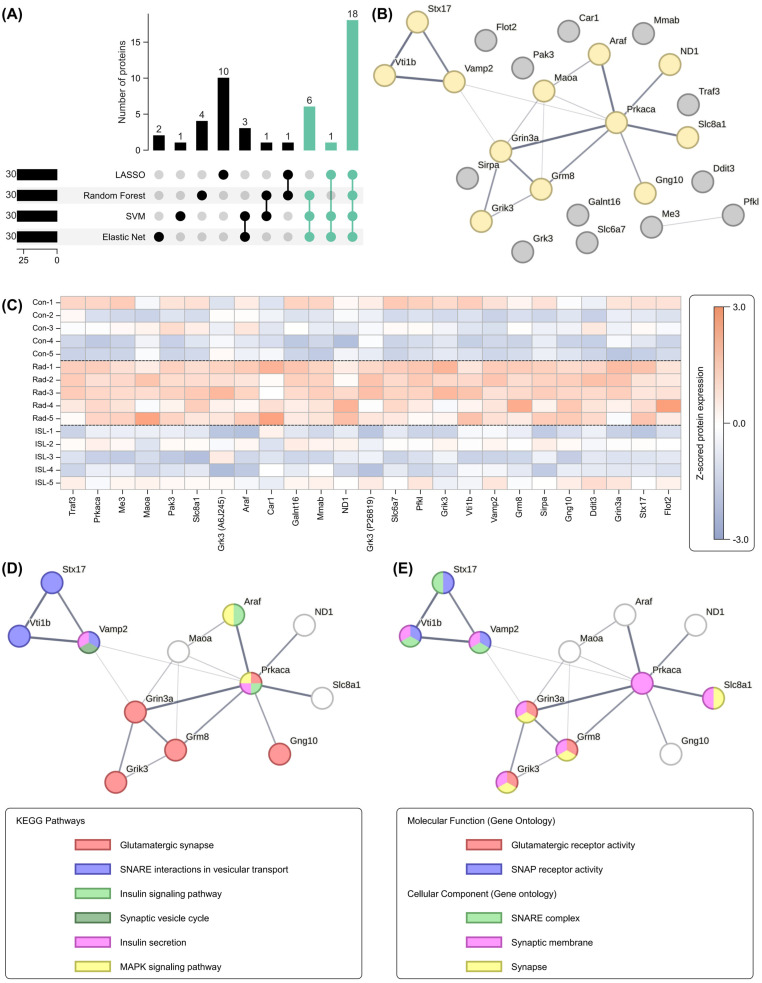
Identification of key ISL radiation mitigation targets. (**A**) A total of 25 ISL radiation mitigation-related proteins identified by at least three models (ISL–RAD). (**B**) PPI network for the 25 ISL radiation mitigation-related proteins (with confidence score threshold of 0.300 in STRGIN database). (**C**) Heatmap for the 25 proteins across samples in CON, RAD, and ISL groups. (**D**) Enriched KEGG pathways for the 25 selected proteins. Disconnected nodes are hidden. (**E**) Enriched GO pathways for the 25 selected proteins. Disconnected nodes are hidden.

Consensus analysis across the above-mentioned four machine learning models revealed 25 ISL radiation mitigation-related proteins (6 + 1 + 18) consistently identified by at least 3 machine learning models (Figure 8A). A protein–protein interaction (PPI) network was constructed for these 25 proteins (Figure 8B), and a z-scored heatmap of their expression is presented in Figure 8C. Overall, these 25 proteins exhibited upregulation in the RAD group and subsequently returned to levels comparable to the CON group with treatment of the ISL group. Using the STRING database, the PPI network highlighted a sub-cluster comprising 12 proteins with interactions: Stx17, Vti1b, Vamp2, Araf, Maoa, Prkaca, ND1, Slc8a1, Grin3a, Grik3, Grm8, and Gng10, as highlighted in Figure 8B with the detailed fold change and *p*-value for RAD–CON and ISL–RAD comparison shown in Table 1.

Functional enrichment analysis revealed predominantly enriched pathways centered on neuronal communication. KEGG pathway enrichment analysis (Figure 8D) shows highly significant involvement in glutamatergic synapse regulation (Grin3a, Gng10, Grm8, Prkaca, Grik3), SNARE-mediated vesicular transport (Stx17, Vamp2, Vti1b), and synaptic vesicle cycling (Vamp2). This synaptic focus was further corroborated by convergent enrichment in insulin and MAPK signaling pathways—including insulin signaling (Prkaca, Araf), insulin secretion (Prkaca, Vamp2), and MAPK cascade activation (Prkaca, Araf). Furthermore, Gene Ontology analysis (Figure 8E) reinforced these findings through localization patterns, with profound gene enrichment at synaptic structures: synapse (Grin3a, Grik3, Grm8, Slc8a1), synaptic membrane (Grin3a, Grm8, Grik3, Vti1b, Vamp2, Prkaca, Slc8a1), and SNARE complexes (Stx17, Vamp2, Vti1b). Molecular functions were similarly dominated by glutamate receptor activity (Grin3a, Grm8, Grik3) and SNAP receptor functionality (Stx17, Vamp2, Vti1b). These results establish that ISL’s radiation mitigation mechanism fundamentally involves the coordinated modulation of synaptic architecture and glutamate-mediated neurotransmission, integrated with metabolic, vesicular trafficking machinery and stress response-related pathways.

KEGG analysis identified the “glutamatergic synapse” as the most significantly enriched pathway, involving five proteins (Grin3a, Gng10, Grm8, Prkaca, Grik3). GO molecular function analysis specifically highlighted “glutamate receptor activity”, exclusively attributed to Grin3a, Grm8, and Grik3, within the 12-protein cluster, defining their role in glutamate binding and signal initiation. GO cellular component analysis further revealed significant enrichment at the “synaptic membrane”, where the glutamate receptors Grik3, Grm8, and Grin3a are integral postsynaptic membrane proteins. This localization positions them as the primary receptors for synaptic glutamate signaling and makes them directly accessible to potential modulators ISL. In contrast, proteins such as Prkaca, Araf, Ddit3, Maoa, and the SNARE components (Stx17, Vamp2, Vti1b) function primarily within the cytoplasm or on intracellular vesicles, representing downstream effectors or supporting machinery rather than the primary signal receptors. Therefore, based on the KEGG and GO enrichment results, the glutamate receptors Grik3, Grm8, and Grin3a were identified as the most three probable targets for ISL’s radiation mitigation.

**Figure 9:**
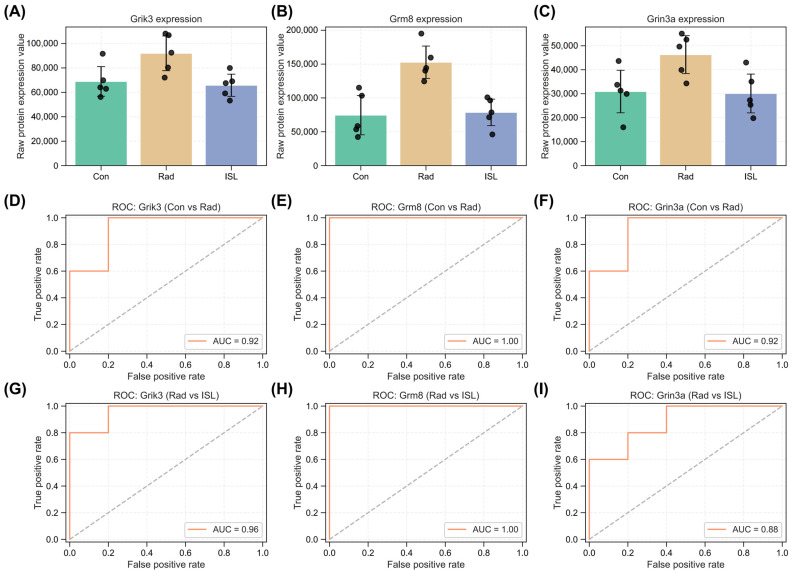
Proteomic analysis of glutamate receptors Grik3, Grm8, and Grin3a. (**A**–**C**) Raw protein expression values for Grik3 (**A**), Grm8 (**B**), and Grin3a (**C**) across CON, RAD, and ISL groups, with individual data points shown as black dots. All bar graphs display mean ± standard deviation (*n* = 5). (**D**–**I**) Receiver operating characteristic (ROC) curves for distinguishing groups. (**D**) (Grik3, CON vs. RAD), (**E**) (Grm8, CON vs. RAD), (**F**) (Grin3a, CON vs. RAD), (**G**) (Grik3, RAD vs. ISL), (**H**) (Grm8, RAD vs. ISL), and (**I**) (Grin3a, RAD vs. ISL) show ROC curves.

Proteomic validation was focused on the candidate glutamate receptors Grik3, Grm8, and Grin3a. Radiation exposure significantly increased their expression compared to CON groups, and ISL normalizing levels to near control values (Figure 9A–C). ROC analysis demonstrated excellent discriminatory power for these receptors. AUC values were near-perfect or excellent for differentiating CON vs. RAD (Grik3: 0.92; Grm8: 1.00; Grin3a: 1.00; Figure 9D–F) and RAD vs. ISL groups (Grik3: 0.96; Grm8: 1.00; Grin3a: 0.88; Figure 9G–I). These results validate Grik3, Grm8, and Grin3a as primary targets for ISL’s radiation mitigation and align with their enrichment in the glutamatergic synapse pathway.

### 2.7. Validation of Interactions Between Isoliquiritigenin and Grik3, Grm8, Grin3a

**Figure 10:**
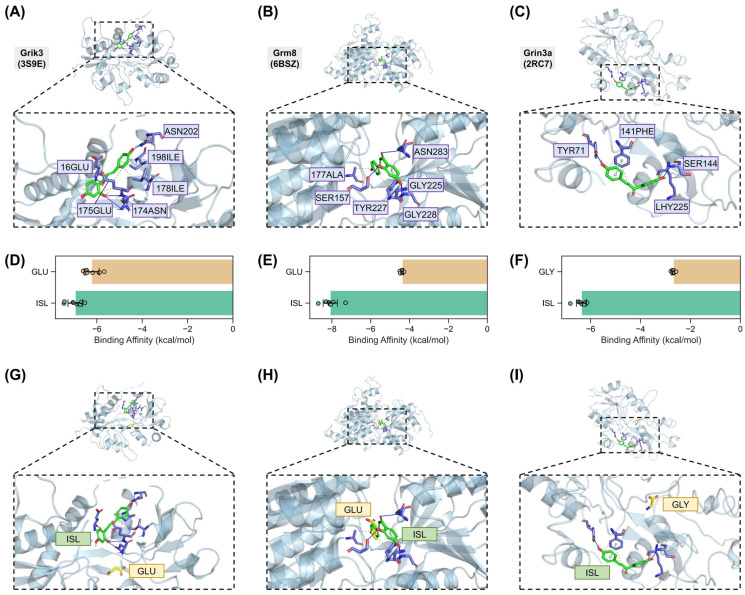
Validation of interactions between ISL and Grik3, Grm8, and Grin3a with molecular docking. (**A**–**C**) Molecular docking between ISL and Grik3 (**A**), Grm8 (**B**), and Grin3a (**C**). (**D**–**F**) ISL’s binding affinity with Grik3 (**D**), Grm8 (**E**), and Grin3a (**F**) compared to native ligands. Individual data points shown as dots. All bar graphs display mean ± standard deviation (*n* = 9). (**G**–**I**) ISL’s binding pose and native ligand’s binding pose of Grik3 (**G**), Grm8 (**H**), and Grin3a (**I**).

To validate the potential binding of ISL to the identified glutamate receptors, molecular docking and molecular dynamics simulations were performed (Table 2). Molecular docking analysis revealed interactions between ISL and each receptor. For Grik3 (PDB: 3S9E, Figure 10A), ISL formed extensive hydrophobic interactions with GLU175 (distances: 3.40 Å, 3.87 Å), ILE178 (3.63 Å, 3.65 Å), and ILE198 (3.56 Å), complemented by hydrogen bonds with GLU16 (1.85 Å), ASN174 (3.47 Å), GLU175 (2.34 Å), and ASN202 (2.57 Å). In Grm8 (Figure 10B), hydrophobic interactions were observed with ALA177 (3.81 Å) and TYR227 (3.87 Å, 3.99 Å), while hydrogen bonds involved SER157 (2.09 Å), GLY225 (2.55 Å), TYR227 (3.24 Å), GLY228 (1.93 Å), and ASN283 (3.16 Å). For Grin3a (Figure 10C), hydrophobic interactions occurred with PHE141 (3.67 Å, 3.57 Å) and LYS225 (3.98 Å), alongside hydrogen bonds with TYR71 (3.20 Å, 2.87 Å) and SER144 (1.94 Å), and a prominent π-π stacking interaction with PHE141 (3.82 Å). These molecular docking results indicate the stable binding of ISL with all of the identified three glutamate receptors.

ISL’s binding affinity was further compared to native ligands (glutamate for Grik3, Grm8; glycine for Grin3a). For Grik3, ISL exhibited stronger binding affinity (mean: −6.95 ± 0.32 kcal/mol) compared to glutamate (−6.23 ± 0.33 kcal/mol). Similarly, for Grm8, ISL demonstrated superior affinity (−6.36 ± 0.20 kcal/mol) over glutamate (−2.68 ± 0.06 kcal/mol). In Grin3a, ISL also showed enhanced binding (−8.09 ± 0.37 kcal/mol) relative to the native ligand glycine (−4.38 ± 0.07 kcal/mol) (Figure 10D–F). These data confirm ISL’s high-affinity interactions with three receptors, supporting its potential as a competitive ligand.

Comparative analysis of binding poses revealed distinct spatial- and residue-specific interactions for different receptors. For Grm8, ISL and the native ligand glutamate shared overlapping binding pockets, with both engaging ALA177 (hydrophobic) and adjacent residues (Figure 10H). Grik3 and Grin3a displayed divergent binding modes. ISL occupied distinct sub-pockets compared to glutamate, engaging non-overlapping residues (ISL: GLU175, ILE178, ASN202 vs. glutamate: THR92, THR144, ARG97 in Grik3; ISL: PHE141, LYS225, SER144 vs. glutamate: SER123, SER125, ARG130 in Grin3a) (Figure 10G–I and Table S1). This residue-level divergence suggests that ISL modulates receptor activity through different binding mechanisms in Grik3 and Grin3a.

To further validate the interactions between ISL and receptors, molecular dynamics (MD) simulations were conducted for 100 ns based on the most stable docking conformations. MD simulation serves as a powerful tool to explore the dynamic behavior and stability of ligand–receptor complexes over time, providing insights into conformational changes, interaction, and overall complex robustness under near-physiological conditions.

**Figure 11:**
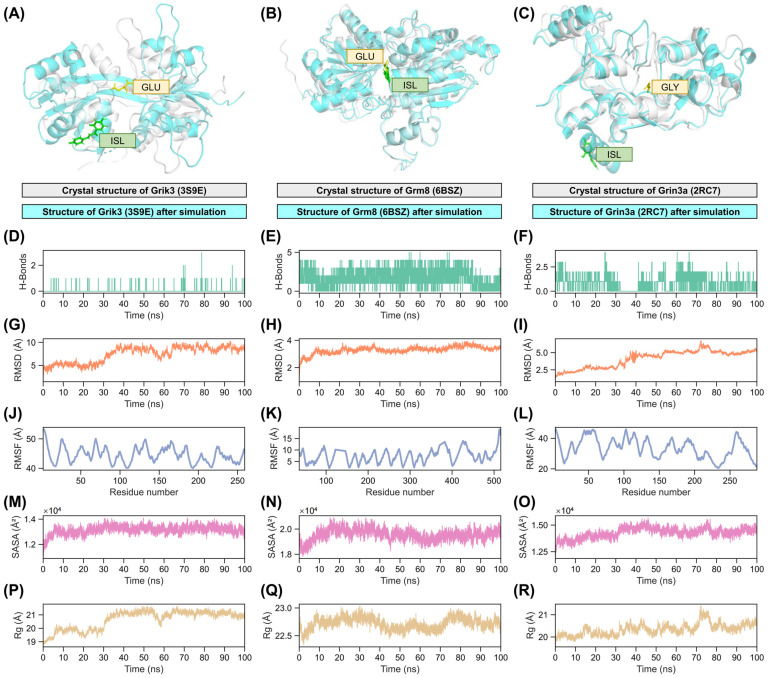
Validation of interactions between ISL and Grik3, Grm8, and Grin3a with molecular dynamic simulation. (**A**–**C**) Crystal structure, structure after simulation, binding pose of the native ligand, and ISL’s binding pose after dynamic simulation. For (**A**), the receptor is Grik3 (3S9E) with GLU as the native ligand. For (**B**), the receptor is Grm8 (6BSZ) with GLU as the native ligand. For (**C**), the receptor is Grin3a (2RC7) with GLY as the native ligand. (**D**–**F**) Hydrogen bond (H-bond) counts between the receptor and ISL during the 100 ns simulation for Grik3 (**D**), Grm8 (**E**), and Grin3a (**F**). (**G**–**I**) Root mean square deviation (RMSD) of the receptor- ISL complex during simulation for Grik3 (**G**), Grm8 (**H**), and Grin3a (**I**). (**J**–**L**) Root mean square fluctuation (RMSF) of the receptor per residue for Grik3 (**J**), Grm8 (**K**), and Grin3a (**L**). (**M**–**O**) Solvent-accessible surface area (SASA) of the receptor- ISL complex during simulation for Grik3 (**M**), Grm8 (**N**), and Grin3a (**O**). (**P**–**R**) Radius of gyration (Rg) of the receptor–ISL complex during simulation for Grik3 (**P**), Grm8 (**Q**), and Grin3a (**R**).

Structural alignment analysis of the simulated receptor–ligand complexes revealed deviations from the original crystal structures (Figure 11A–C). For all three receptors, notable structural differences were observed between the crystal structures and the MD-equilibrated complexes, indicating that ISL binding induces conformational adjustments. The relative positions of ISL and the native ligands (glutamate for Grik3/Grm8; glycine for Grin3a) were examined. In the case of Grm8, ISL occupied a binding pocket closely overlapping with its native ligand glutamate, consistent with a shared binding mode. Conversely, for both Grik3 and Grin3a, ISL and the respective native ligands (glutamate and glycine, respectively) were situated in distinct, spatially separated locations within the binding site. This observation aligns with the results predicted by molecular docking.

Hydrogen bond (H-bond) dynamics were quantified throughout the simulation trajectory (Figure 11D–F). Stable H-bond patterns are indicative of persistent and specific ligand–receptor interactions. Grik3 maintained an average of 1–3 H-bonds with ISL, reflecting moderate but consistent stability. Grin3a exhibited a higher and more stable H-bond count, predominantly ranging between two and four bonds, suggesting stronger and more persistent polar interactions. Grm8 demonstrated the most robust H-bonding, frequently maintaining three to five bonds, indicating a highly stable polar network supporting ISL binding.

Root mean square deviation (RMSD) analysis of the backbone atoms provides a measure of overall conformational stability relative to the starting structure (Figure 11G–I). Lower RMSD values generally indicate greater stability. Grik3-ISL complex exhibited the highest RMSD, fluctuating significantly between 5 and 10 Å, suggesting more substantial conformational rearrangements during the simulation period. Grin3a-ISL showed moderate conformational adjustments with RMSD values slightly higher, averaging around 2.5–5 Å. Notably, the Grm8-ISL complex displayed stable dynamics with an average RMSD fluctuating minimally less than 4 Å, indicative of a stable complex conformation. For 80ns–100ns, the fluctuation range of the RMSD curves of the complexes corresponding to the three receptors was basically maintained within 2 Å. This means that MD has entered a stable stage, reflecting that ISL and the three receptors can bind relatively stable.

Root mean square fluctuation (RMSF) per residue assesses local flexibility, particularly highlighting regions like binding sites (Figure 11J–L). Lower fluctuations suggest greater rigidity. Grik3 demonstrated overall low residue flexibility, with RMSF values mostly below 10 Å, indicating a well-ordered complex. Similarly, most residues in the Grm8 complex fluctuated below 10 Å, signifying the most stable binding environment in comparison with the others. Grin3a, however, displayed notably higher fluctuations, particularly around the binding site residues where varying peaks reached up to 20 Å. This increased flexibility suggests lower adaptability within the Grin3a binding pocket when complexed with ISL.

Solvent-accessible surface area (SASA) was monitored to evaluate changes in solvent exposure of the complex, which relates to ligand desolvation and hydrophobic packing stability (Figure 11M–O). Grik3 maintained a relatively constant SASA ranging from 12,000 to 14,000 Å^2^, indicating consistent exposure and stable hydrophobic core packing. The SASA profile for Grm8 was stable, fluctuating about 18,000–22,000 Å^2^ Grin3a showed slightly higher solvent exposure, with SASA values about 12,500–15,500 Å^2^, potentially correlating with the observed higher conformational flexibility and most stable binding.

Finally, the radius of gyration (Rg) was employed to assess the structural compactness of the receptor–ISL complexes (Figure 11P–R). Lower Rg values indicate tighter molecular packing. The Grik3-ISL complex demonstrated consistent compactness, with Rg values stabilizing at 19–21 Å. The Grm8-ISL complex maintained high structural integrity, exhibiting minimal fluctuation in Rg (22.5–23.0 Å), which represents the most stable configuration among the three complexes. In contrast, Grin3a-ISL showed slightly reduced compactness, with Rg values ranging between 20 and 21 Å, correlating with its higher RMSD and SASA values observed earlier. Notably, all three complexes maintained Rg variations within a narrow 2 Å range, confirming the overall stability of ISL binding.

In summary, molecular docking followed by 100 ns molecular dynamics simulations robustly validate interactions between ISL and the glutamate receptors Grik3, Grm8, and Grin3a. The observed binding stabilities, hydrogen bonding networks, and overall complex stability across multi metrics confirm stable ISL binding to all three receptors. Notably, although ISL binds to all three receptors, it exhibits distinct binding modes. For Grm8, the binding site coincides with that of the native ligand. However, for the other two receptors (Grik3 and Grin3a), the binding sites do not coincide with their respective native ligands, indicating that ISL likely interacts with Grik3 and Grin3a at alternative sites.

## 3. Discussion

Radiotherapy for brain and neck tumors often damages healthy tissue, leading to cognitive decline and life-threatening complications like cerebral necrosis [8]. Despite advanced techniques such as proton therapy, repeated sessions can still cause cumulative radiation injury, significantly impairing patients’ quality of life [36]. To evaluate ISL’s radioprotective efficacy, we compared it with four glutamate modulators. Glutamate receptor agonist CHPG and PAM CDPPB are known to enhance glutamatergic signaling and reduce excitotoxic glutamate accumulation [37]. Glutamate receptor antagonists DL-AP3 and NAM CTEP suppress receptor activity and may exacerbate glutamate dysregulation [38]. Our cellular studies demonstrated that glutamate receptor agonists CHPG and PAM CDPPB attenuated radiation-induced damage, partially improving cell viability, preserving membrane integrity, mitigating ATP depletion, and reducing excitotoxic glutamate elevation. In contrast, antagonists DL-AP3 and NAM CTEP failed to protect or exacerbated injury across these parameters. ISL exhibited radiation mitigation at the cellular level, effectively restoring viability to near-normal levels, completely recovering ATP concentrations, and substantially preserving membrane integrity. Furthermore, ISL normalized glutamate homeostasis to baseline and reduced radiation-induced ROS accumulation.

Glutamate, the primary excitatory neurotransmitter in the CNS, plays pivotal roles in synaptic transmission, plasticity, and cognitive function [39]. Its signaling is tightly regulated through interactions with ionotropic (NMDA, AMPA) and metabotropic (mGluR) receptors [40]. Moreover, astrocytes dynamically maintain extracellular glutamate homeostasis via glutamate reuptake and the glutamate–glutamine cycle [41]. It is also reported that dysregulation of glutamate homeostasis, characterized by pathological accumulation, triggers excitotoxicity [42]. This process involves sustained receptor overactivation, mitochondrial dysfunction, and energy depletion [43]. These reported glutamate-related findings were corroborated in our cellular experiments on ISL’s radioprotective effects.

Building upon cellular findings, ISL also demonstrated neuronal radiation mitigation efficacy in vivo. Behavioral assessments revealed that ISL attenuated radiation-induced hippocampal damage and associated functional deficits. Furthermore, ISL ameliorated radiation-triggered anhedonia, evidenced by restored sucrose preference. ISL preserved neuronal architecture and synaptic integrity histologically, suppressing neuroinflammation by IL-1β reduction, restoring PSD-95 synaptic marker, and maintaining dendritic spine density particularly plasticity-critical stubby and filopodium-like subtypes. These results confirm ISL’s capacity to mitigate radiation injury at the organismal level, preserving both hippocampal structure and affective function.

Given the constraint of sample size (*n* = 5 per group [44,45]) in proteomics studies, any single computational method is bound to have its own limitation. For example, while WGCNA is a powerful tool for identifying co-expression modules, its reliability and stability are highly sensitive to sample size. The resulting network modules may be more susceptible to noise and outliers. Therefore, our study employs a multi-step integrated computational pipeline to identify and validate key targets of ISL. By sequentially combining DEPs identification, enrichment analysis, WGCNA, machine learning algorithms, molecular docking, and dynamics simulations, the methodology substantially mitigates the risk of overfitting, a critical advantage given the high-dimensional nature of proteomic data and the limited sample size. Four-dimensional-DIA proteomic profiling of rat brain tissues was performed to comprehensively assess ISL’s radiation mitigation related regulation. KEGG pathway analysis revealed that ISL-mediated radiation mitigation significantly enriched pathways involved in the synaptic vesicle cycle, glutamatergic synapse, MAPK signaling pathway, and SNARE interactions in vesicular transport. Gene Ontology (GO) analysis further corroborated these findings. ISL radiation mitigation related DEPs clustered around synaptic transport and glutamatergic synapse. Overall, the pathway enrichment highlighted a critical overlap in glutamatergic signaling and synaptic vesicle pathways.

**Figure 12:**
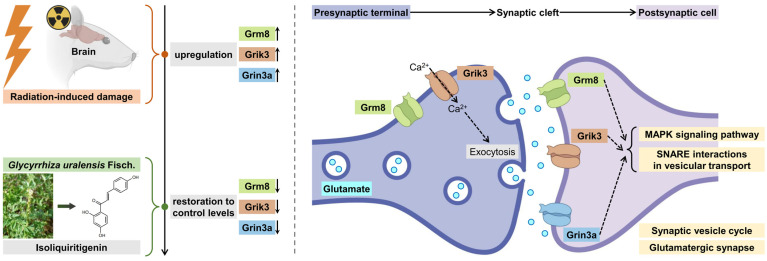
Isoliquiritigenin as a potent natural radiation mitigant that modulates glutamate homeostasis and synaptic plasticity primarily through targeting Grik3, Grm8, and Grin3a.

In order to further explore the ISL’s core targets related to radiation mitigation, an integrated ensemble model (differentially expressed protein identification, pathway enrichment analysis, WGCNA, machine learning, and PPI) was used. Twelve key proteins were identified: Araf, Gng10, Grik3, Grin3a, Grm8, Maoa, Mt-nd1, Prkaca, Slc8a1, Stx17, Vamp2, and Vti1b. Functional clustering revealed their collective convergence on glutamate homeostasis and synaptic plasticity regulation. Grik3 and Grin3a directly modulate ionotropic glutamate receptor signaling [46]. The metabotropic receptor Grm8 further fine-tunes synaptic glutamate levels via presynaptic inhibition [47], with its activation reducing vesicular release [48]. This mechanism also exhibits protein–protein interaction with SNARE-mediated exocytosis (with Vamp2, Stx17, and Vti1b) [49]. Furthermore, Maoa indirectly sustains glutamate equilibrium by catabolizing monoamines that compete with glutamate reuptake transporters [50]. Slc8a1 (NCX1) maintains Ca^2+^ homeostasis essential for NMDA receptor gating and prevents Ca^2+^-induced mitochondrial dysfunction [51]. Signal transduction mediators Araf (MAPK pathway), Prkaca (PKA catalytic subunit) [52], and Gng10 (Gβγ subunit) bridge receptor activation to downstream plasticity effectors [53]; PKA phosphorylates AMPA/NMDA receptors to modulate LTP [54] and Gng10 is reported to enable G-protein beta-subunit binding activity [55]. Notably, Grik3, Grm8, and Grin3a are enriched on the “glutamatergic synapse” pathway “glutamate receptor activity”. They are localized to the “synaptic membrane”, positioning them as the primary receptors directly accessible to ISL. Therefore, glutamate receptors Grik3, Grm8, and Grin3a are regarded as the key targets for ISL’s radiation mitigation, as shown in Figure 12.

Overall, ISL is a naturally occurring flavonoid found in foods such as licorice. It exhibits a broad pharmacological spectrum and functions as a natural radio-modulatory agent. By targeting glutamate receptors such as Grm8, Grik3, and Grin3a, ISL modulates synaptic function and glutamate homeostasis, highlighting ISL as a novel natural product candidate for mitigating radiation-induced neural damage during radiotherapy.

## 4. Materials and Methods

### 4.1. Cellular Studies

#### 4.1.1. Cellular Culture and Treatment

The HT22 mouse hippocampal neuronal cell line (obtained from Qingqi (Shanghai) Biotechnology Development Co., Ltd., Shanghai, China) was cultured in high glucose Dulbecco’s Modified Eagle Medium (catalog number: 11995065, Thermo Fisher Scientific, Waltham, MA, USA) supplemented with 10% heat-inactivated fetal bovine serum (catalog number: ST30-3302, PAN Biotech UK Ltd., Gateshead, UK), 1% non-essential amino acids (catalog number: 11140050, Thermo Fisher Scientific, Waltham, MA, USA), and 1% penicillin/streptomycin (catalog number: 15140122, from Thermo Fisher Scientific, Waltham, MA, USA). Cells were maintained at 37 °C in a humidified incubator with 5% CO_2_ (model Heracell VIOS 250i CR, Thermo Fisher Scientific, Waltham, MA, USA). The medium with the above components is referred to as complete medium. Culture medium was replaced every 72 h.

For experimental procedures, HT22 cells were seeded in 96-well plates at a density of 6 × 10^4^/100 μL per well and incubated overnight at 37 °C to ensure complete cell adherence. Cells were then pretreated for 24 h with the following compounds dissolved in DMSO (final concentration ≤ 0.1% *v*/*v*, catalog number: MKL-T884153, Meryer Co., Ltd., Shanghai, China) at maximum non-toxic dose. The final concentration of each compound was as follows: ISL (catalog number: MKL-I811912, Meryer Co., Ltd., Shanghai, China) 10 µM; DL-AP3 (catalog number: MKL-D911393, Meryer Co., Ltd., Shanghai, China) 10 µM; CTEP (catalog number: MKL-C796017, Meryer Co., Ltd., Shanghai, China) 2 nM; CHPG (catalog number: MKL-C995824, Meryer Co., Ltd., Shanghai, China) 50 µM; CDPPB (catalog number: MKL-C918576, Meryer Co., Ltd., Shanghai, China) 30 nM. Following treatment with compounds for 24 h, the irradiated group received ^60^Coγ radiation at a dose rate of 1 Gy/min for 2 min, resulting in a total dose of 2 Gy [56,57] at the Cobalt Source Room, Beijing Normal University, China (machine: ^60^Co radiation machine, model: 0800574, Beijing, China). Immediately after irradiation, the culture medium was replaced with fresh medium to remove harmful metabolites (e.g., free radicals) generated upon radiation. Control groups received no irradiation; all other procedures were the same as the irradiated groups. All assays were performed 24 h post-irradiation [58].

#### 4.1.2. Cell Viability Assay (CCK-8)

HT22 cells were seeded in 96-well plates (catalog number: ASO-CC-9534-01, Meryer Co., Ltd., Shanghai, China) at a density of 6000 cells/well in 100 μL complete medium and incubated overnight (at 37 °C) for attachment. After 24 h post-radiation incubation, cellular viability was assessed using the CCK-8 kit (catalog number CA1210, Beijing Solarbio Science & Technology Co., Ltd., Beijing, China) following the kit instructions. Cell viability was calculated as a percentage relative to the absorbance (using microplate reader, model CYTATION3, BioTek Instruments, Inc., Winooski, VT, USA) of the non-irradiated control group.

#### 4.1.3. Lactate Dehydrogenase (LDH) Measurement

Following a 24 h post-radiation incubation, lactate dehydrogenase was evaluated using a commercial lactate dehydrogenase assay kit (catalog number C0017, Beyotime Biotechnology, Beijing, China) following the kit instruction (25 °C). This assay was performed on the same set of cells used for the cell viability assay (three replicates). Absorbance was measured (microplate reader, model CYTATION3 from BioTek Instruments, Inc., Winooski, VT, USA) at 490 nm (primary signal) with a reference wavelength of 600 nm to correct for background interference.

#### 4.1.4. ATP Quantification

Following a 24 h post-radiation incubation, intracellular ATP levels were quantified using a commercial ATP detection kit (catalog number S0026, Beyotime Biotechnology, Beijing, China) following kit instructions. An ATP standard curve was prepared by serially diluting the ATP standard solution in lysis buffer. For measurement, 100 μL of ATP detection working solution was added per well of an opaque microplate and equilibrated at room temperature for 3–5 min. Subsequently, 20 μL of sample or standard was added to each well, mixed immediately, and luminance intensity was measured after a minimum 2 s delay using a luminometer (microplate reader with luminescence module, model CYTATION3 from BioTek Instruments, Inc., Winooski, VT, USA). ATP concentrations were calculated from the standard curve and normalized to total protein concentration (determined separately), using a bicinchoninic acid (BCA) assay kit (catalog number P0012, Beyotime Biotechnology, Beijing, China). All procedures were performed on ice or at 4 °C to maintain ATP stability.

#### 4.1.5. Measurement of Glutamate

Intracellular glutamate concentration was quantified 24 h post-radiation using a commercial glutamate content assay kit (model BC1585 from Beijing Solarbio Science & Technology Co., Ltd., Beijing, China). Cells were collected. Culture medium was completely aspirated from the culture plate without disturbing the cell monolayer. The cells were washed with pre-cooled sterile PBS (1×, pH 7.4) to remove residual serum and metabolites, after which the PBS was thoroughly aspirated. An appropriate volume of trypsin-EDTA (0.25%) (catalog number: MKL-T917516, Meryer Co., Ltd., Shanghai, China) was added to cover the cell layer, followed by incubation at 37 °C for 1–2 min until cell rounding and detachment were observed under a microscope (Nikon, ECLIPSE E100, Tokyo, Japan). Digestion was terminated by adding complete medium supplemented with serum, and the cells were gently pipetted to generate a single-cell suspension. The suspension was transferred to a centrifuge tube and centrifuged at 1000 rpm for 5 min at 4 °C. Finaly, the supernatant was discarded, and the cell pellet was retained for further processing. Cell pellets were resuspended in Reagent One in the kit (1 mL per 10 million cells). Cell lysis was achieved by ultrasonication (model S-250D from Branson Ultrasonics Co., Danbury, CT, USA). Lysates were centrifuged (10,000 rpm, 10 min, room temperature). The supernatant was collected for analysis. For the assay, 40 μL of sample (or standard) was added to wells of a 96-well UV plate, followed by 160 μL Reagent Three in the kit and 10 μL Reagent Four in the kit. After gentle mixing, the absorbance at 340 nm was measured immediately (using the microplate reader, model CYTATION3 from BioTek Instruments, Inc., Winooski, VT, USA) (A1 at 20 s) and after 5 min 20 s (A2). According to the kit instructions, a standard curve was constructed using known concentrations of glutamate standard included in the kit. Glutamate concentration was calculated based on the change in absorbance (ΔA = A1 − A2) and comparison to standard curve, reflecting radiation-induced alterations in glutamate metabolism.

#### 4.1.6. Reduced Glutathione (GSH) Quantification

Intracellular reduced glutathione (GSH) levels were quantified using a commercial GSH content assay kit (catalog number JYM1319Ra, Wuhan ColorfulGene Biological Technology Co., Ltd., Wuhan, China). Twenty-four hours post-radiation treatment, cells were collected following the procedure above, washed twice with PBS (600× *g*, 10 min) (catalog number: M52268, Meryer Co., Ltd., Shanghai, China), and resuspended in 1 mL Reagent One in the kit. Cells were lysed by ultrasonication (model S-250D from Branson Ultrasonics Co., Danbury, CT, USA). The lysate was centrifuged (8000× *g*, 10 min) and the supernatant was collected for analysis. In a 96-well plate, 100 μL of sample (or standard), 700 μL Reagent Two in the kit, and 200 μL Reagent Three in the kit were combined per well. After thorough mixing and a 2 min incubation at room temperature, absorbance was measured at 412 nm using a spectrophotometer (model CYTATION3, BioTek Instruments, Inc., Winooski, VT, USA).

#### 4.1.7. Measurement of Reactive Oxygen Species (ROS)

To measure the intracellular ROS levels, a commercial DCFH-DA probe (Solarbio, Beijing, China) was used. For this, 6 h after radiation, a concentration of 10 μmol/L DCFH-DA was added to the treated HT22 cells, which were then incubated in the dark at 37 °C for 30 min. After incubation, the cells were washed three times with PBS to remove any unbound probe. Fluorescence imaging was performed using a Nikon A1R confocal microscope (with an inverted Nikon Ti-E body, a perfect focus system, and a 5% CO_2_ incubation system for live-cell imaging, Tokyo, Japan). Images were acquired with a Plan Apo VC 60× Oil objective (NA 1.4, Tokyo, Japan), utilizing confocal mode with identical settings across all samples: a 488 nm laser for excitation at a fixed intensity (50 mW, unvaried between acquisitions) and emission detection at 525 ± 25 nm through a GaAsP PMT detector (Tokyo, Japan) (configured for FITC/DCF detection with a standard filter set: Ex 460–480 nm, Em 495–540 nm). The pinhole size, laser power (2.5%), gain (65%), and offset (0) were rigorously kept constant. Image resolution was set to 1024 × 1024 pixels, and the optical zoom was fixed at 1× to maintain consistent field size. Fluorescence intensity quantification was performed using NIS-Elements software (Nikon, Tokyo, Japan), with background subtraction from PBS-washed, unlabeled control cells.

### 4.2. Animal Studies

#### 4.2.1. Animal Model of Radiation

Wistar wild-type male rats (5–6 weeks) were supplied by SiBeiFu Biotechnology Co., Ltd., Beijing, China. Upon arrival, rats were maintained in a sterile housing condition at 25 °C, with a 12 h light/dark cycle and rats had free access to food and water. This study was approved by the Animal Care and Utilization Committee of Beijing Institute of Technology (Beijing, China) (Project No. BIT-EC-SCXK2019-0010-R-011).

After three days of adaptive feeding, 15 healthy male rats were randomly divided into three groups, with each group having 5 rats. The control group (CON) rats were given 10 mL/kg distilled water containing 0.5% sodium carboxymethyl cellulose (catalog number: M88146, Meryer Co., Ltd., Shanghai, China) daily to the day of sacrifice. The whole-brain irradiated group (RAD) rats were given 10 mL/kg distilled water containing 0.5% sodium carboxymethyl cellulose daily to the day of sacrifice on the third day after irradiation. In the ISL intragastric administration group (ISL), rats received a daily dose of 10 mL/kg fresh ISL aqueous suspension (2 mg/mL) [59] starting 2 h post-irradiation and continuing until the third day after irradiation. ISL suspension was made with 0.5% sodium carboxymethyl cellulose aqueous solution for immediate use. Upon irradiation, a lead brick (5 cm) was used to shield the lower body from the neck of the rats, exposing the head of the rat to radiation. Under radiation exposure, the rats, under anesthesia with 0.2 mL of a 10% chloral hydrate solution (catalog number: MKL-H765693, Meryer Co., Ltd., Shanghai, China) per 100 g administered intraperitoneally, were hung vertically. The depth of anesthesia was confirmed by the absence of response to a foot pinch. The head was aligned with the radiation beam window (machine: ^60^Co radiation machine, model: 0800574, Beijing, China) and 60Coγ were irradiated at a time with a source distance of 200 cm. A dose rate of 2 Gy/min and a total dose of 30 Gy [32,33,34] was used at the Cobalt Source Room, Beijing Normal University, China. Rats in the CON group were also anesthetized with 0.2 mL of a 10% chloral hydrate solution (catalog number: MKL-H765693, Meryer Co., Ltd., Shanghai, China) and suspended in the same vertical position for the same duration as the RAD group, but did not receive any irradiation. After the irradiation, the experimental animals were transported back to the animal housing and continued normal feeding.

#### 4.2.2. Sucrose Preference Test

The sucrose preference test (SPF) was performed on each group of rats immediately after radiation. Rats were individually caged in a quiet, soundproof room before the test. On the first day, rats were habituated with 1% sucrose (catalog number: MKL-S982424, Meryer Co., Ltd., Shanghai, China) for 24 h. On the second day, the rats were habituated with two bottles, one with 1% sucrose and the other with pure water. For the next 24 h, the rats were not given food or water. During the last 1hr, the rats were given 1% sucrose and pure water. Sucrose preference percentage (%) was calculated following the Formula (1):

Sucrose preference percentage (%) = sucrose intake (mL)/total intake (mL) × 100%.(1)

#### 4.2.3. Histopathological Analysis

The rats were sacrificed on the third day after irradiation, and the rat brains were excised and completely immersed in 4% paraformaldehyde (Beijing Solarbio Science & Technology Co., Ltd., Beijing, China) for histopathological analysis. The brain tissue was fixed for 24–26 h, dehydrated, and embedded in paraffin. Standard histological processing was performed; tissues were dehydrated through a graded ethanol series (SCRC, 100092683, Shanghai, China) using a dehydrator (DIAPATH, Donatello, Montichiari, Italy), cleared in xylene (SCRC, 10023418, Shanghai, China) or Environmentally Friendly Dewaxing Transparent Liquid (Servicebio, G1128-1L, Wuhan, China), and embedded in paraffin using an embedding machine (Wuhan Junjie, JB-P5, Wuhan, China). Paraffin blocks were sectioned at 4 μm thickness with a rotary microtome (Shanghai Leica, RM2016, Shanghai, China), mounted on adhesive slides (Servicebio, G6012-1, Wuhan, China), and air-dried. For H&E staining, sections were deparaffinized and rehydrated, then stained with hematoxylin (Servicebio, G1076, Wuhan, China) for 5 min, differentiated, blued, and counterstained with eosin for 5 s. Dehydration was performed through graded ethanol and normal butanol (SCRC, 100052190, Shanghai, China), followed by xylene clearance and mounting with neutral gum (SCRC, 10004160, Shanghai, China) and cover glasses (Citotest, 10212432C, Jiangsu, China). Processed sections were examined under an upright optical microscope (Nikon, ECLIPSE E100, Tokyo, Japan) equipped with an imaging system (Nikon, DS-U3, Tokyo, Japan) to assess cell morphology, inflammatory infiltration, and other pathological features.

To assess the radiation-induced histopathological damage and the radiation mitigation efficacy of ISL, scoring was employed by three independent pathologists blinded to the experimental groups (service provided by Wuhan Servicebio Technology Co., Ltd., Wuhan, China). The evaluation was performed on hematoxylin and eosin (H&E)-stained sections focusing on the overall hippocampal formation, and specific subregions including the CA2 area and the dentate gyrus (DG). The scoring criteria were based on the extent of structural disorganization, neuronal pyknosis, eosinophilia, vacuolation, and reduction in neuronal density. Each parameter was graded on a scale of 0 to 3. 0, normal morphology; 1—mild changes; 2—moderate damage; and 3—severe histopathological alterations. The final score for each image was derived from the average of scores assigned by the three evaluators. For quantitative analysis of neuronal density in the hemibrain hippocampal formation, the total number of neurons in the hippocampus of each hemibrain section was counted using the Image-Pro Plus 6.0 analysis software (Bethesda, MD, USA).

#### 4.2.4. Measurement of Inflammatory Factors IL-1β in the Brain Tissue

The level of IL-1β in the rat brain tissue was determined by the commercial enzyme-linked immunosorbent assay (ELISA) kit (catalog number: YX-091201R from Shanghai Guduo Biotechnology Co., Ltd., Shanghai, China), following the instructions of the ELISA kits.

#### 4.2.5. Golgi-Cox Staining Analysis

A sharp blade was used to cut a 5–10 mm thick tissue block from the hippocampus of the fixed rat brain tissue. The tissue block to be observed was then quickly rinsed with distilled water to remove any blood. The rat brain tissue block was placed in the G1069-30ML (Wuhan Service Bio Technology, Wuhan, China) bottle. The staining fixative was poured into a special waste liquid bucket in the fume hood. It was replaced with the Golgi staining (Wuhan, China) solution to completely immerse the tissue block, which was then placed in a cool and ventilated place at 26 °C and away from light for a total of 14 days. After an immersion period of 48 h, the staining solution was replaced with a new solution; subsequent replacements occurred every 3 days. After staining was completed, the staining solution was poured into a special waste liquid bucket in the fume hood. The tissue was then treated with a new tissue treatment solution after 1 h of treatment and treated at 4 °C and away from light for 3 days. The brain tissue was attached to the tray of the vibration slicer (Wuhan, China) using 502 super glue and immersed in the tissue treatment solution. Sections were cut at 60 μm and, after cutting, were transferred to a slide with the help of a brush. After picking up the slice, tissue treatment solution was added onto its surface, and it was left to dry for a short while before development. The slice was washed with ultrapure water, developed with Golgi developer (Wuhan, China) for 30 min, and then washed with water. Any excess water on the slide was carefully wiped off, and the slice was finally sealed with glycerol gelatin.

#### 4.2.6. RT-qPCR Analysis

Total RNA was extracted from brain tissue using the reagent kit (catalog number R0024, Beyotime Biotechnology, Beijing, China), following the kit’s instructions. The resulting RNA samples were subsequently reverse transcribed into complementary DNA using the reagent kit (model M-MLV, Beijing Solarbio Science & Technology Co., Ltd.). The RT-qPCR was performed by real-time PCR (IQ5, Bio-Rad) utilizing GAPDH as a housekeeping gene. The expression of each gene was normalized to that of glyceraldehyde-3-phosphate dehydrogenase (GAPDH). The forward primer sequence of PSD95 was AGACGGTGACGCAGATGGAA. The reverse primer sequence of PSD95 was TCGGGGAACTCGGAGAGAAG. The forward primer sequence of GAPDH was CTGGAGAAACCTGCCAAGTATG. The reverse primer sequence of GAPDH was GGTGGAAGAATGGGAGTTGCT. The relative expression levels (fold change) of each target gene were normalized to that of GAPDH mRNA levels in each sample using the 2^−∆∆CT^ method. Each experiment was conducted with three biological replicates to guarantee independent measurements.

#### 4.2.7. 4D-DIA Proteomic Analysis

- 4D-DIA proteomics data acquisition

Tissue samples were individually pulverized in liquid nitrogen. The resulting powder was lysed using SDT buffer (containing 100 mM NaCl) supplemented with 1/100 volume DTT, followed by ultrasonication (model S-250D from Branson Ultrasonics Co., Danbury, CT, USA) on ice for 5 min. Lysates were incubated at 95 °C for 8–15 min, immediately cooled on ice for 2 min, and centrifuged at 12,000× *g* for 15 min at 4 °C. The supernatant was collected and alkylated with iodoacetamide (IAM) for 1 h at 25 °C in the dark. Proteins were precipitated by thoroughly vortexing the alkylated lysate with 4 volumes of pre-cooled acetone, followed by incubation at −20 °C for a minimum of 2 h. After centrifugation (12,000× *g*, 15 min, 4 °C), the pellet was collected, washed once with 1 mL cold acetone, and subsequently dissolved completely in Dissolution Buffer (DB buffer).

Protein concentration was determined using a Bradford assay kit (model P0006 from Beyotime Biotechnology, Beijing, China). A standard curve was generated using bovine serum albumin (BSA) solutions ranging from 0 to 0.5 g/L. Both BSA standards and diluted sample solutions (20 µL final volume per well) were loaded in triplicate onto a 96-well plate. Following the rapid addition of 180 µL G250 dye reagent to each well, the plate was incubated at 25 °C for 5 min. Absorbance was measured at 595 nm, and sample protein concentrations were calculated based on the standard curve. For quality assessment, 20 µg of each protein sample was resolved by SDS-PAGE using a 12% polyacrylamide gel. Electrophoresis was performed at 120 V through the stacking gel (20 min) and 150 V through the resolving gel (50 min). Proteins were visualized by staining with Coomassie Brilliant Blue R-250 reagent and destained until bands were clearly defined.

Protein samples were adjusted to a final volume of 100 µL with DB lysis buffer (8 M urea, 100 mM triethylammonium bicarbonate [TEAB], pH 8.5). Samples were digested with trypsin at 37 °C for 4 h, followed by a second addition of trypsin and overnight digestion. The digestion was terminated by acidification with formic acid to pH less than 3. Acidified samples were centrifuged at 12,000× *g* for 5 min at 25 °C. The supernatant was loaded onto a C18 solid-phase extraction column. Peptides were washed three times with washing buffer (0.1% formic acid, 3% acetonitrile). Peptides were eluted with elution buffer (0.1% formic acid, 70% acetonitrile). Eluted peptides were collected and lyophilized.

Lyophilized peptides were reconstituted in 10 µL of mobile phase A (0.1% formic acid in water). After centrifugation at 14,000× *g* for 20 min at 4 °C, 200 ng of peptide (based on pre-digestion quantification) was injected. Chromatographic separation was performed on a Vanquish Neo UHPLC system (Thermo Scientific) equipped with a C18 pre-column (Acclaim PepMap 100, 5 mm × 300 μm, 5 μm) maintained at 50 °C and an analytical column (PepMap Neo UHPLC C18, 150 μm × 15 cm, 2 μm). Mobile phases consisted of A (0.1% formic acid in water) and B (0.1% formic acid in 80% acetonitrile).

The elution gradient begins at time zero (0 min) with a flow rate of 2.5 µL/min and a mobile phase composition of 96% A and 4% B. At 0.2 min, the flow rate decreases to 1.3 µL/min while maintaining the same mobile phase ratio (96% A/4% B). At 0.3 min, the flow rate further decreases to 0.8 µL/min as the composition shifts to 92% A and 8% B, remaining stable at these conditions until 0.5 min. Between 0.5 min and 14.2 min, the flow rate holds at 0.8 µL/min while the mobile phase gradually changes to 77.5% A/22.5% B at the 14.2 min mark. By 21.1 min, still at 0.8 µL/min, the composition reaches 65% A/35% B. Subsequently, at 21.5 min, the flow rate increases to 2.5 µL/min concurrent with a shift to 45% A/55% B and initiation of a Column Wash step. At 21.9 min, the mobile phase rapidly transitions to 1% A/99% B while maintaining the 2.5 µL/min flow rate, and this condition persists until the run stops at 22.6 min.

Eluting peptides were analyzed using a Thermo Orbitrap Astral mass spectrometer (Waltham, MA, USA) equipped with an Easy-Spray (ESI) ion source. The key mass spectrometry parameters were as follows: spray voltage—2.0 kV; ion transfer tube temperature—290 °C; full MS1 scan range—*m*/*z* 380–980; MS1 resolution—240,000 (@ *m*/*z* 200); MS1 automatic gain control (AGC) target—500%; Data-Independent Acquisition (DIA) mode with 300 variable windows (2 Th width); normalized collision energy (NCE)—25%; MS2 scan range—*m*/*z* 150–2000; MS2 resolution (Astral analyzer)—80,000; maximum MS2 injection time—3 ms. (.raw) files were generated.

- Quality control

The mass spectrometry data files were processed using Proteome Discoverer software (version 2.1) for database searching. Searches were performed against the UniProtKB database restricted to the species Rattus norvegicus. A false discovery rate threshold set at less than 1% was applied for peptide spectrum matches, peptides, and proteins. Proteins with at least 4 missing values in any single group (CON/RAD/ISL) were removed. Proteins exhibiting at least 3 missing values in at least two groups were excluded.

- Differential expression analysis for proteomic

Differential expression analysis was performed using UniProt IDs as identifiers derived from database searching. Homogeneity of variance between groups was evaluated using Levene’s test with an alpha level of 0.01. Proteins exhibiting a Levene’s *p*-value exceeding 0.01 were considered to have equal variances. An independent *t*-test was conducted. A dual-threshold criterion defined statistically significant differential expression: (1) a protein expression fold change threshold of 1.2 and (2) a *p*-value threshold of 0.05. Proteins were classified as differentially expressed only when meeting both criteria simultaneously.

- Pathway enrichment analysis

Functional enrichment analysis was conducted using the STRING platform (version 12.0). This analysis utilized the STRING API and Python (version 3.10.16). The species was specified as Rattus norvegicus, identified by NCBI taxon ID 10116. The STRING enrichment method was employed with default parameter settings. For proteins intended for enrichment analysis, UniProt IDs were converted to gene names using the online UniProt ID mapping tool (https://www.uniprot.org/id-mapping, accessed on 20 December 2024). Then, the STRING ID mapping tool was employed to convert gene names or protein descriptions into standardized STRING query items. For a given protein list, the STRING API returns enrichment results including Gene Ontology (biological processes, molecular functions, cellular components) and KEGG pathways. Pathway enrichment significance was determined using a false discovery rate threshold of 0.05.

#### 4.2.8. Weighted Gene Correlation Network Analysis (WGCNA)

Weighted gene correlation network analysis (WGCNA) was performed using the OmicVerse package [60] (version 1.7.1) in Python (version 3.10.16). WGCNA is applied to identify modules of highly correlated proteins associated with experimental conditions (RAD or ISL). Missing values in the RAD and ISL groups (5 replicates each) were imputed using group-specific means. Following data preprocessing, the top 5000 most variable proteins based on median absolute deviation were selected for network construction. A signed adjacency matrix was generated using a soft-thresholding power (β = 10) determined via scale-free topology analysis (power range from 1 to 15), followed by calculation of a topological overlap matrix to minimize noise. Dynamic hybrid tree-cutting with a DeepSplit of 2 identified co-expression modules, whose “eigengenes” parameters were computed for downstream analysis. Module–trait associations and *p*-value were calculated by default parameters. The module with *p*-value less than 0.05 was identified as the key module.

#### 4.2.9. Four Machine Learning Models for Identifying Key Proteins

To identify key proteins distinguishing radiation-induced damage and ISL-related radiation mitigation, four machine learning models (LASSO, Random Forest, Linear Support Vector Machine, Elastic Net logistic regression, as outlined below) were implemented using scikit-learn (version 1.5.2) in Python (version 3.10.16). Therefore, the computational tasks for the machine learning models are formulated as binary classification tasks. For RAD (*n* = 5) versus CON (*n* = 5) classification and ISL (*n* = 5) versus RAD (*n* = 5) classification, features were standardized to zero mean and unit variance using the StandardScaler function in scikit-learn. All models employed fixed random states and balanced class weights.

LASSO (least absolute shrinkage and selection operator)-regularized logistic regression was applied for feature selection using L1 penalty. The model was configured with an inverse regularization strength C = 10,000 (minimal regularization), ‘liblinear’ solver, and maximum iterations = 10,000. Proteins with non-zero coefficients were sorted and selected.

Random Forest was used for non-linear feature importance assessment. A total of 1000 trees with ‘sqrt’ mode ‘max_features’ and Gini impurity splitting criterion are included. Feature importance was calculated as mean Gini impurity reduction across all trees.

Linear Support Vector Machine (SVM) with L2 penalty was implemented for maximum margin separation. The regularization parameter C was set as 0.1. The regularization parameter-controlled margin strictness, with a linear kernel for interpretable coefficients. Feature weights were extracted from the primal hyperplane coefficients.

Elastic Net logistic regression combined L1 and L2 penalties (L1_ratio was set as 0.01) using the ‘saga’ settings. The regularization parameter C was set as 0.1. Max_iter was set as 10,000. This balanced feature selection and coefficient stability (correlation handling).

The absolute values of signed coefficients in LASSO, 0–1 scaled Gini importance scores in Random Forest, signed hyperplane coefficients in SVM, and signed coefficients in Elastic Net are defined as coefficient or importance. Proteins were ranked by the absolute values per model–task combination. The top 30 proteins are selected for each model.

#### 4.2.10. Protein–Protein Interaction Network Analysis

The protein–protein interaction network was constructed using the STRING database (version 12.0, accessed on 20 December 2024). The analysis was restricted to the target species Rattus norvegicus (taxonomic identifier: 10116). Functional and physical protein associations were retrieved. A minimum interaction confidence score threshold of 0.300 was applied.

### 4.3. Molecular Docking

Molecular docking was performed using AutoDock Vina [61] (v1.2.3). The crystal structure of 3 glutamate receptors (PDBID: 2RC7 for Grin3a, 3S9E for Grik3, 6BSZ for Grm8) were obtained from the Protein Data Bank. The 3D conformer of ligand (isoliquiritigenin) was downloaded from PubChem. Both the receptor and ligand files were converted to .pdbqt format using OpenBabel [62] (v3.1.1). The geometric center of ISL was aligned to (12.58, 48.09, 7.86) for Grin3a, (2.69, −29.42, −14.12) for Grik3, and (−37.12, −22.33, 31.60) for Grm8. The geometric center was determined as the geometric center of glutamic acid in Grm8 and Grik3’s crystal structure, and the geometric center of glycine in Grin3a’s crystal structure. Molecular docking was conducted using a 30 Å × 30 Å × 30 Å grid box centered at the geometric center. After scoring and energy minimization, semi-flexible docking was performed with an exhaustiveness value of 200 and the other parameters defaulted. The ten most stable binding poses were generated with predicted binding affinities for each receptor–ligand complex. The default scoring function in AutoDock Vina was applied. PLIP [63] (Protein–Ligand Interaction Profiler) was applied to identify the interactions between receptor and ligand based on docking results. PyMol (open-source, version 3.1.5.1) was applied to visualize the binding pose and interactions. 

### 4.4. Molecular Dynamic Analysis 

The protein–ligand complex was prepared using the Amber LEaP module [64] with the ff14SB force field and TIP3P water model. An octahedral water box was defined with a 10.0 Å buffer, and Na^+^/Cl^−^ ions were incorporated. Energy minimization was performed: (1) 2000 steps with 50 kcal/mol·Å^2^ constraints on protein backbone atoms (CA, C, O, N of residues 1–630); (2) 1000 steps with constraints reduced to 14 kcal/mol·Å^2^; (3) 2000 steps at 4 kcal/mol·Å^2^ constraints; and (4) 4000 unconstrained steps, using a 10.0 Å non-bonded cutoff with steepest descent algorithm. The system was equilibrated under NVT conditions for 50 ps while heating from 0.1 K to 300.00 K with 2 kcal/mol·Å^2^ constraints on all protein atoms, followed by 50 ps of NPT density equilibration at 300.00 K and 1 bar. MD was conducted for 100 ns (50,000,000 steps, 2 fs timestep) in the NPT ensemble without constraints, with temperature maintained via Langevin dynamics (γ = 1.0 ps^−1^) and pressure regulated by a Berendsen barostat (τ = 2.0 ps). Trajectories were saved per 10 ps and analyzed using CPPTRAJ [65] to compute RMSD, Rg, SASA, and RMSF.

### 4.5. Statistic Methods

The experimental results were expressed as mean ± standard deviation (*n* = 5 for proteomic analysis [66,67] and *n* = 3 for other cellular and animal experiments). The data of each group were statistically processed by GraphPad Prism software (version 10.1.2). An independent sample *t*-test was used to determine whether the difference between groups was statistically significant. The use of the independent *t*-test was justified as the primary data were assumed to be approximately normally distributed based on the central limit theorem, given the nature of the measured biological variables. Formal tests for normality were not conducted due to the small sample size. The *t*-test is considered robust to mild deviations from normality. Homoscedasticity was assumed for the standard *t*-test.

## 5. Conclusions

This study systematically investigated the neuronal radiation mitigation related effects of ISL against radiation-induced neuronal damage using a multi-modal experimental approach. Key findings demonstrate ISL’s radiation mitigation potential across biological scales. In vitro analyses revealed that ISL treatment markedly reduced radiation-induced cytotoxicity, as evidenced by decreased LDH leakage and ROS generation, while restoring cellular ATP levels and preventing pathological glutamate accumulation. In vivo validation showed ISL administration effectively attenuated radiation-related anhedonia behaviors and preserved hippocampal synaptic integrity in rodent models. High-throughput proteomic profiling identified multiple pathways modulated by ISL, mainly including synaptic vesicle recycling, glutamatergic transmission, MAPK cascade, SNARE-mediated exocytosis, and insulin signaling networks. Through integrated proteomics and AI-driven analysis, ISL’s core targets—glutamate receptors Grik3, Grm8, and Grin3a—were identified, with molecular docking and molecular dynamics simulations demonstrating ISL’s superior binding stability at these receptor sites compared to endogenous ligands. In summary, these results establish ISL as a promising natural therapeutic candidate with multi-target efficacy against radiotherapy-induced neural injury, acting through synergistic modulation of excitotoxicity, oxidative stress, and synaptic maintenance pathways.

## Figures and Tables

**Table 1:** Fold change and *p*-value for 12 key proteins in RAD–CON and ISL–RAD comparison.

UniprotID	Protein	FC (RAD–CON)	*p*-Value (RAD–CON)	FC (ISL–RAD)	*p*-Value (ISL–RAD)
A6JZR8	Araf	1.21679	0.02708	0.63141	0.00354
Q3KRE3	Gng10	1.99501	0.00005	0.55094	0.00182
P42264	Grik3	1.33416	0.03918	0.71525	0.01468
Q9R1M7	Grin3a	1.49793	0.03213	0.65006	0.02106
P70579	Grm8	2.04935	0.00319	0.51491	0.00137
A0A8I6AT92	Maoa	1.31883	0.02602	0.70331	0.00989
P21396	Maoa	1.32761	0.01922	0.73558	0.01890
P03889	Mt-nd1	1.23889	0.01227	0.82758	0.01090
A0A8I5YBQ9	Prkaca	1.49942	0.01078	0.69403	0.00127
A6H9P0	Slc8a1	1.53984	0.01354	0.58458	0.00404
Q9Z158	Stx17	1.26830	0.00966	0.75174	0.00198
P63045	Vamp2	1.57046	0.00297	0.76330	0.01232
P58200	Vti1b	1.23527	0.01934	0.82430	0.00324

**Table 2:** Interactions between ISL and Grik3, Grm8, and Grin3a.

Receptor	Interaction	Amino Acid	Distance (Å)	H-A Distance (Å)
Grik3	Hydrophobic interactions	175 GLU	3.40	
	Hydrophobic interactions	175 GLU	3.87	
	Hydrophobic interactions	178 ILE	3.63	
	Hydrophobic interactions	178 ILE	3.65	
	Hydrophobic interactions	198 ILE	3.56	
	Hydrogen Bonds	16 GLU		1.85
	Hydrogen Bonds	174 ASN		3.47
	Hydrogen Bonds	175 GLU		2.34
	Hydrogen Bonds	202 ASN		2.57
Grm8	Hydrophobic interactions	177 ALA	3.81	
	Hydrophobic interactions	227 TYR	3.87	
	Hydrophobic interactions	227 TYR	3.99	
	Hydrogen Bonds	157 SER		2.09
	Hydrogen Bonds	225 GLY		2.55
	Hydrogen Bonds	227 TYR		3.24
	Hydrogen Bonds	228 GLY		1.93
	Hydrogen Bonds	283 ASN		3.16
Grin3a	Hydrophobic interactions	141 PHE	3.67	
	Hydrophobic interactions	141 PHE	3.57	
	Hydrophobic interactions	225 LYS	3.98	
	Hydrogen Bonds	71 TYR		3.20
	Hydrogen Bonds	71 TYR		2.87
	Hydrogen Bonds	144 SER		1.94
	PI stacking	141 PHE	3.82	

## Data Availability

The data presented in this study are available upon reasonable request from the corresponding author.

## Supplementary Materials

The following supporting information can be downloaded at https://www.mdpi.com/article/10.3390/ph18091307/s1: Figure S1: Cytotoxicity of compounds in HT22 cells; Figure S2: Scatter of samples in different groups based on principal component analysis; Figure S3: Heatmap of ISL radiation mitigation-related differentially expressed proteins; Figure S4: Parameter search for radiation related WGCNA; Table S1: Interactions between Grik3, Grm8, and Grin3a with native ligands. Table S2: Effect sizes and confidence intervals of significance test in cellular studies. Table S3: Effect sizes and confidence intervals of significance test in animal studies. Table S4: Stability testing results of machine learning models for the top 30 features.
