# Supplementary material for: Isoliquiritigenin as a Neuronal Radiation Mitigant: Mitigating Radiation-Induced Anhedonia Tendency Targeting Grik3/Grm8/Grin3a via Integrated Proteomics and AI-Driven Discovery
Source: Pharmaceuticals (Basel). 2025 Aug 30;18(9):1307. doi: 10.3390/ph18091307 (PMC12472569; doi:10.3390/ph18091307)
Supplement: Supplementary file 1 [file pharmaceuticals-18-01307-s001.zip › pharmaceuticals-3807881-supplementary.pdf]

# Supplementary Information

# Content

## Supplementary Figures

**Figure S1.** Cytotoxicity of compounds in HT22 cells.

**Figure S2.** Scatter of samples in different groups based on principal component analysis.

**Figure S3.** Heatmap of ISL radiation mitigation-related differentially expressed proteins.

**Figure S4.** Parameter search for radiation related WGCNA analysis.

## Supplementary Tables

**Table S1.** Interactions between Grik3, Grm8, Grin3a with native ligands.

**Table S2.** Effect sizes and confidence intervals of significance test in cellular studies.

**Table S3.** Effect sizes and confidence intervals of significance test in animal studies.

**Table S4.** Stability testing results of machine-learning models for the top 30 features.

## Supplementary Figures

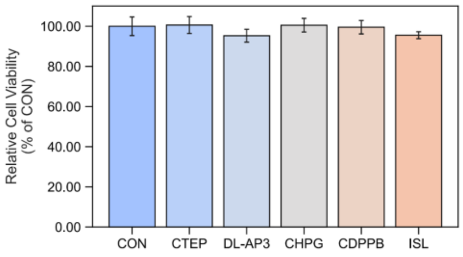


**Figure S1.** Cytotoxicity of compounds in HT22 cells.

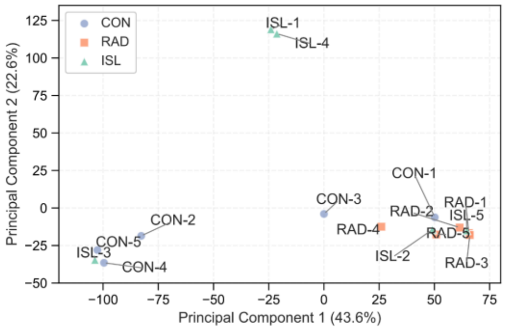


**Figure S2.** Scatter of samples in different groups based on principal component analysis.

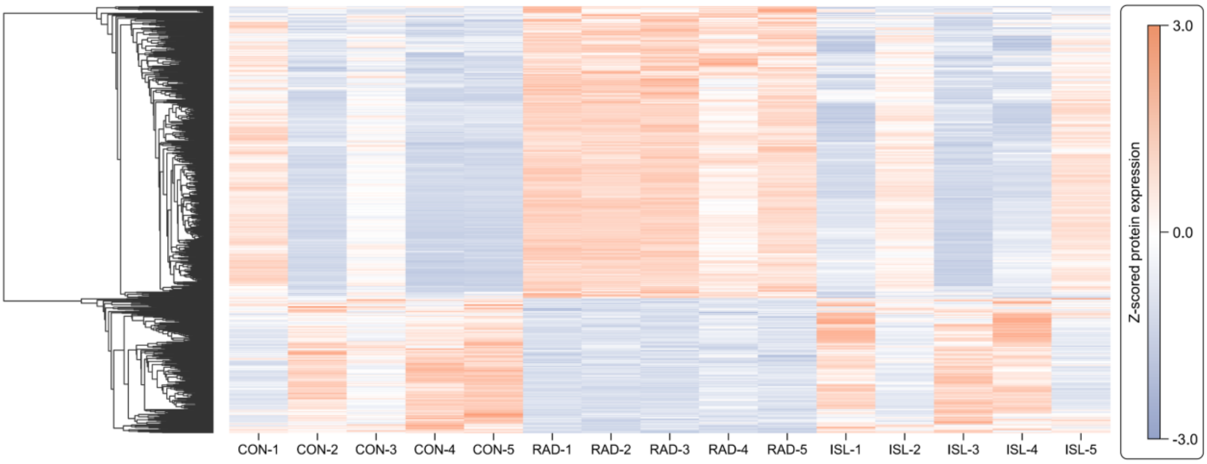


**Figure S3.** Heatmap of ISL radiation mitigation-related differentially expressed proteins.

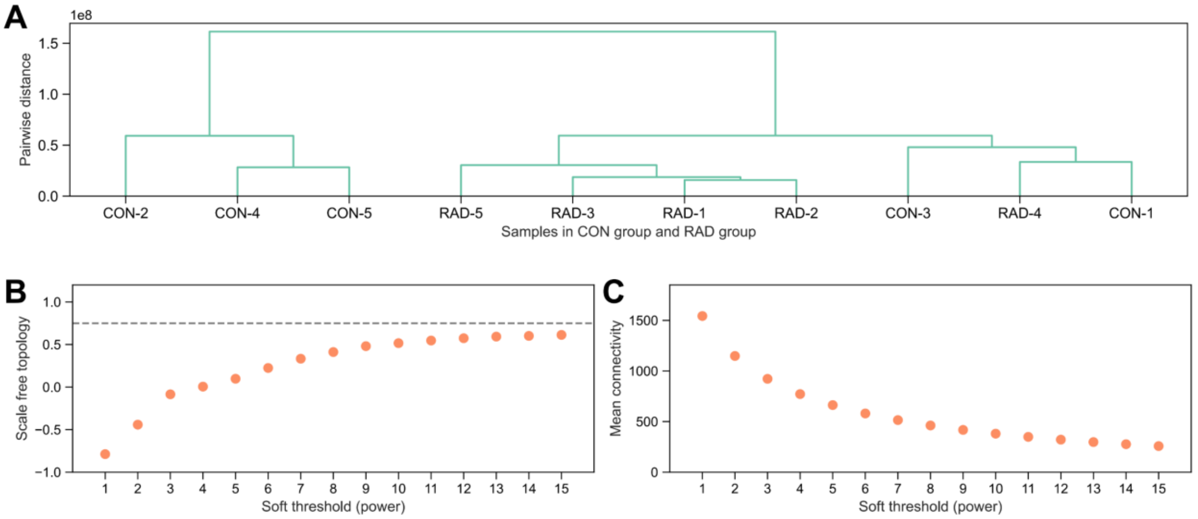


**Figure S4.** Parameter search for radiation related WGCNA analysis. **A.** Hierarchical clustering of samples in CON group and RAD group. **B.** Scale free topology under different soft-thresholding parameters. **C.** Mean connectivity under different soft-thresholding parameters.

## Supplementary Tables

**Table S1.** Interactions between Grik3, Grm8, Grin3a with native ligands.

| Receptor | Interaction | Amino Acid | Distance(Å) | H-A Distance(Å) |
|---|---|---|---|---|
| Grik3 | Hydrophobic interactions | 63 TYP | 3.56 | |
| | Hydrophobic interactions | 259 GLU | 1.51 | |
| | Hydrogen Bonds | 90 PRO | | 2.75 |
| | Hydrogen Bonds | 92 THR | | 1.93 |
| | Hydrogen Bonds | 92 THR | | 2.20 |
| | Hydrogen Bonds | 143 ALA | | 1.93 |
| | Hydrogen Bonds | 144 THR | | 2.02 |
| | Hydrogen Bonds | 144 THR | | 1.72 |
| | Hydrogen Bonds | 259 GLU | | 2.95 |
| | Hydrogen Bonds | 259 GLU | | 2.37 |
| | Water Bridges | 139 VAL | 3.88 | |
| | Water Bridges | 139 VAL | 2.81 | |
| | Water Bridges | 142 GLY | 2.75 | |
| | Water Bridges | 145 MET | 3.98 | |
| | Water Bridges | 191 GLU | 2.74 | |
| | Salt Bridges | 97 ARG | 3.42 | |
| Grm8 | Hydrophobic interactions | 177ALA | 3.94 | |
| | Hydrophobic interactions | 601GLU | 1.53 | |
| | Hydrogen Bonds | 156SER | | 2.12 |
| | Hydrogen Bonds | 179THR | | 1.99 |
| | Hydrogen Bonds | 179THR | | 2.52 |
| | Hydrogen Bonds | 309ASP | | 1.83 |
| | Hydrogen Bonds | 601GLU | | 2.21 |
| | Salt Bridges | 71LYS | 4.63 | |
| | Salt Bridges | 75ARG | 3.49 | |
| | Salt Bridges | 314LYS | 3.75 | |
| | Salt Bridges | 401LYS | 3.65 | |
| Grin3a | Hydrogen Bonds | 123 SER | | 1.87 |
| | Hydrogen Bonds | 125 SER | | 1.79 |
| | Hydrogen Bonds | 180 SER | | 2.42 |
| | Water Bridges | 181 ALA | 3.83 | |
| | Water Bridges | 181 ALA | 4.09 | |
| | Salt Bridges | 130 ARG | 3.47 | |

**Table S2.** Effect sizes and confidence intervals of significance test in cellular studies.

| | Comparison | Cohen_d | CI_lower | CI_upper |
|---|---|---|---|---|
| CCK8 | RAD vs CON | -3.8359 | -6.5324 | -1.1393 |
| | ISL vs RAD | 2.3820 | 0.2898 | 4.4743 |
| | DL-AP3 vs RAD | -3.9009 | -6.6271 | -1.1746 |
| | CTEP vs RAD | -2.1738 | -4.1922 | -0.1555 |
| | CHPG vs RAD | 2.2971 | 0.2355 | 4.3587 |
| | CDPPB vs RAD | 0.7335 | -0.9198 | 2.3868 |
| LDH | RAD vs CON | 2.9935 | 0.6633 | 5.3237 |
| | ISL vs RAD | -6.2988 | -10.2055 | -2.3921 |
| | DL-AP3 vs RAD | 0.4214 | -1.1966 | 2.0393 |
| | CTEP vs RAD | 1.0849 | -0.6291 | 2.7989 |
| | CHPG vs RAD | -19.9095 | -31.2874 | -8.5315 |
| | CDPPB vs RAD | -16.8216 | -26.4729 | -7.1703 |
| ATP | RAD vs CON | -19.4123 | -30.5119 | -8.3128 |
| | ISL vs RAD | 12.9834 | 5.4650 | 20.5017 |
| | DL-AP3 vs RAD | 0.5780 | -1.0554 | 2.2114 |
| | CTEP vs RAD | -12.8333 | -20.2686 | -5.3979 |
| | CHPG vs RAD | 20.8879 | 8.9616 | 32.8142 |
| | CDPPB vs RAD | 1.5989 | -0.2394 | 3.4372 |
| Glutamate | RAD vs CON | 4.3930 | 1.4368 | 7.3492 |
| | ISL vs RAD | -3.8526 | -6.5569 | -1.1484 |
| | DL-AP3 vs RAD | -0.9068 | -2.5873 | 0.7738 |
| | CTEP vs RAD | -0.6677 | -2.3120 | 0.9766 |
| | CHPG vs RAD | -4.9083 | -8.1135 | -1.7031 |
| | CDPPB vs RAD | -3.3587 | -5.8431 | -0.8742 |
| GSH | RAD vs CON | -2.4910 | -4.6235 | -0.3585 |
| | ISL vs RAD | 2.3065 | 0.2415 | 4.3715 |
| | DL-AP3 vs RAD | -1.1144 | -2.8344 | 0.6057 |
| | CTEP vs RAD | -1.9300 | -3.8674 | 0.0074 |
| | CHPG vs RAD | 2.0918 | 0.1014 | 4.0823 |
| | CDPPB vs RAD | 11.8392 | 4.9520 | 18.7264 |
| Fluorescence Intensity of ROS | RAD vs CON | 8.1586 | 4.3742 | 11.9431 |
| | ISL vs RAD | -2.5226 | -4.1836 | -0.8616 |

CI: confidence interval.

**Table S3.** Effect sizes and confidence intervals of significance test in animal studies.

| | **Comparison** | **Cohen_d** | **CI_lower** | **CI_upper** |
|---|---|---|---|---|
| Sucrose preference | RAD vs CON | -18.6835 | -29.3751 | -7.9919 |
| | ISL vs RAD | 12.4697 | 5.2351 | 19.7043 |
| IL-1β | RAD vs CON | 30.0503 | 12.9726 | 47.1281 |
| | ISL vs RAD | -36.1469 | -56.6614 | -15.6323 |
| PSD95 | RAD vs CON | -6.9978 | -11.2683 | -2.7272 |
| | ISL vs RAD | 21.3290 | 9.1554 | 33.5027 |
| Stubby spine | RAD vs CON | -7.2618 | -11.6713 | -2.8524 |
| | ISL vs RAD | 6.5629 | 2.5194 | 10.6063 |
| Filopodium-like spine | RAD vs CON | -2.6322 | -4.8184 | -0.4461 |
| | ISL vs RAD | 3.0766 | 0.7120 | 5.4412 |
| Mushroom spine | RAD vs CON | 0.5088 | -1.1172 | 2.1348 |
| | ISL vs RAD | -0.6524 | -2.2948 | 0.9899 |
| Slender spine | RAD vs CON | 0.7646 | -0.8931 | 2.4224 |
| | ISL vs RAD | -0.8812 | -2.5574 | 0.7950 |

CI: confidence interval.

**Table S4. Stability testing results of machine-learning models for the top 30 features.** Each machine-learning model is trained 100 times, with random seed from 0 to 99. Top 30 features of each machine-learning model is recorded. For each feature in the finally selected 25 features, the frequencies in top 30 during the 100 times of training is presented (range from 0 to 100).

| Name | LASSO | RF | SVM | ElasticNet |
|---|---|---|---|---|
| Traf3 | 66 | 66 | 100 | 100 |
| Prkaca | 92 | 100 | 100 | 100 |
| Me3 | 51 | 100 | 100 | 100 |
| Maoa (A0A8I6AT92) | 99 | 68 | 100 | 100 |
| Pak3 | 24 | 100 | 100 | 100 |
| Slc8a1 | 68 | 100 | 100 | 100 |
| Grk3 (A6J245) | 99 | 91 | 100 | 100 |
| Araf | 24 | 100 | 100 | 100 |
| Car1 | 100 | 89 | 100 | 100 |
| Galnt16 | 26 | 82 | 100 | 100 |
| Mmab | 52 | 100 | 100 | 100 |
| ND1 | 59 | 97 | 100 | 100 |
| Grk3 (P26819) | 87 | 62 | 100 | 100 |
| Slc6a7 | 100 | 100 | 100 | 100 |
| Pfkl | 38 | 15 | 100 | 100 |
| Grik3 | 100 | 80 | 100 | 100 |
| Vti1b | 100 | 100 | 100 | 100 |
| Vamp2 | 34 | 100 | 100 | 100 |
| Grm8 | 100 | 100 | 100 | 100 |
| Sirpa | 68 | 87 | 100 | 100 |
| Gng10 | 78 | 100 | 100 | 100 |
| Ddit3 | 93 | 79 | 100 | 100 |
| Grin3a | 51 | 0 | 100 | 95 |
| Stx17 | 77 | 100 | 100 | 100 |
| Flot2 | 95 | 100 | 100 | 100 |
